# Proline-Free Local Turn via N-Oxidation: Crystallographic and Solution Evidence for a Six-Membered N–O⋯H–N Ring

**DOI:** 10.3390/molecules30244676

**Published:** 2025-12-05

**Authors:** Renlin Zheng, Wenjiao Zhao, Shuo Yuan, Tong Wang, Wenyu Lu, Qian Meng, Li Yang, Dequn Sun

**Affiliations:** 1 College of Life Science and Agri-Forestry, Southwest University of Science and Technology, Mianyang 621010, China; zhengrenlin@swust.edu.cn (R.Z.);; 2 Engineering Research Center of Biomass Materials, Ministry of Education, Southwest University of Science and Technology, Mianyang 621010, China; 3 Marine College, Shandong University, Weihai 264200, China

**Keywords:** conformation, restriction, N-oxide, intramolecular hydrogen bonds, peptide

## Abstract

N-oxides are emerging as versatile tools for modulating peptide conformation due to their strong proton-accepting ability and distinct electronic properties. In this study, we report the first crystallographic evidence that an N-oxidized peptide (NOP **5**) containing a proline residue forms an intramolecular six-membered hydrogen bond between the N-oxide oxygen and an adjacent amide proton. This conformational motif is not restricted to proline-containing sequences: NMR spectroscopic analyses (including DMSO-d_6_ titration, VT-NMR, NOE, and concentration-dependent studies) reveal that NOPs **7** and **9**, in which proline is replaced by glycine, adopt the same hydrogen-bonded ring structure in aprotic solvents. Remarkably, this conformation persists even in protic solvent (CD_3_OH), indicating the robustness of the N-oxide-induced hydrogen bond. DFT calculations further support the experimental findings and rationalize the conformational preferences of NOPs **5** and **7**. These results establish N-oxide as a potent and generalizable constraint for stabilizing peptide secondary structures, offering a new strategy for the design of peptidomimetics with tunable rigidity and solvent stability.

## 1. Introduction

Controlling the three-dimensional architecture of peptides is central to the rational design of functional biomolecules and peptidomimetics [1,2,3]. Considerable effort has been devoted to understanding and mimicking native secondary structures, including α-helices, β-sheets, and various turn motifs [4,5,6,7,8]. Beyond natural folds, unnatural foldamers with backbone modifications have been developed to enforce specific conformations through non-covalent interactions [9,10]. The nature and positioning of substituents critically influence the conformational bias of these systems [11].

N-oxides, characterized by a strongly polarized N → O bond, have recently emerged as promising tools for modulating molecular conformation. The oxygen atom of the N-oxide acts as a potent hydrogen-bond acceptor, capable of forming stable intra- or intermolecular interactions with adjacent donor groups [12]. This property has been exploited in crystal engineering, drug design, and catalysis [13,14]. Notably, N-oxides have been employed as pharmaceutical building blocks [11,15], therapeutic agents [16,17], and catalysts in asymmetric synthesis [18,19]. Despite their widespread use, the potential of N-oxides to direct peptide conformation through intramolecular hydrogen bonding remains underexplored.

**Figure 1:**
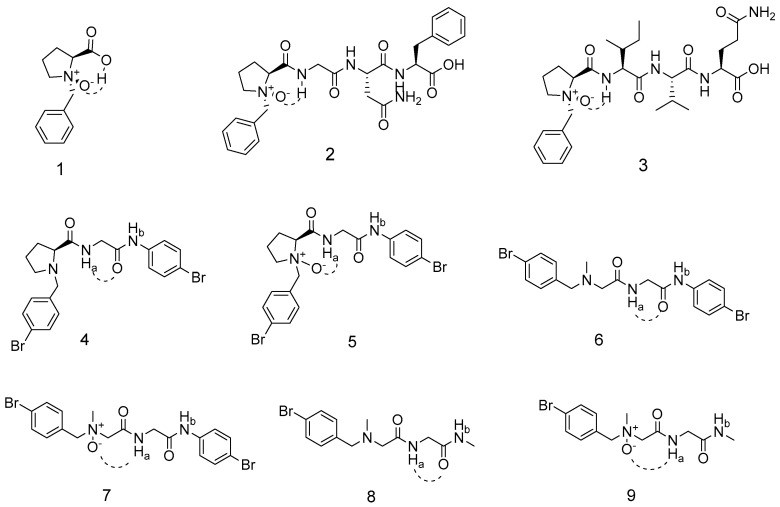
Compound **1**–**3**: the structure of N-oxide compounds containing the proline unit reported by previous papers. Compound **4**–**9**: the structure of normal peptides and N-oxide peptides (NOPs) synthesized in this work.

**Figure 2:**
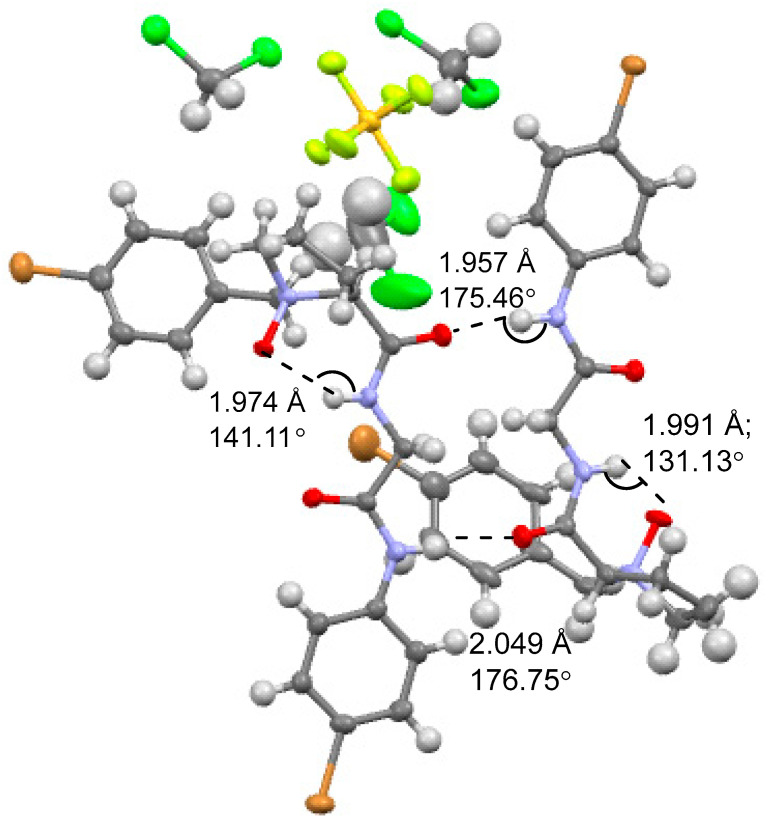
X-ray structure of NOP **5**.

**Figure 3:**
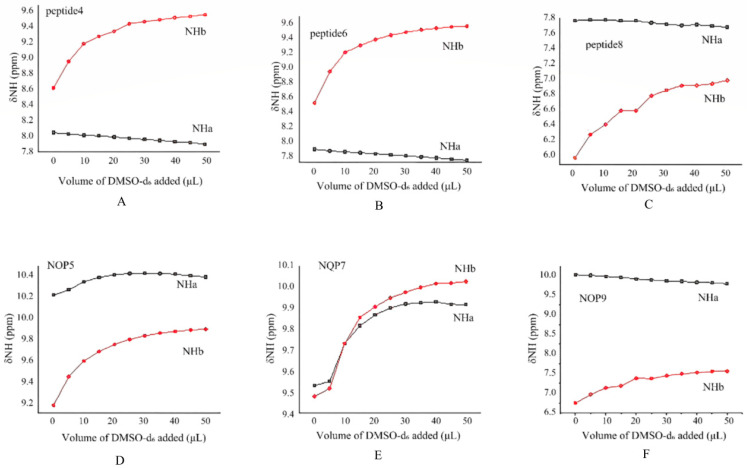
Amide proton chemical shifts plotted as a function of the amount of DMSO-d_6_ added to 10 mM solutions of peptides **4**–**9** (**A**–**F**) in CDCl_3_ (0.5 mL) at room temperature.

Proline is a well-established promoter of peptide folding, frequently nucleating β-turns or α-helices through its rigid pyrrolidine ring [1,20]. Early NMR studies by O’Neil et al. revealed that N-oxide-substituted proline analogs can engage in intramolecular hydrogen bonding in aprotic solvents, but only when proline is present in the sequence (Figure 1, **1**) [21,22]. Building on this, Farahani et al. incorporated N-oxide moieties into short peptides and observed similar hydrogen-bond-mediated constraints (Figure 1, **2** and **3**); however, all constructs retained a proline residue, leaving unresolved whether this residue is required for the observed conformational restriction [23]. Critically, no crystal structure of an N-oxide peptide has been reported to date, and their behavior in protic solvents—where competing intermolecular hydrogen bonding might disrupt the intramolecular motif—remains unexplored.

To resolve the longstanding question of whether proline is required for N-oxide-induced hydrogen bonding, we designed a congeneric series of N-oxide peptides (NOPs) (Figure 1, **4**–**9**) with and without proline, and subjected them to multi-technique conformational analysis. Single-crystal X-ray diffraction of NOP **5** furnished the first atomic-level structure of an N-oxide peptide, revealing an intramolecular six-membered ring secured by an N–O⋯H–N hydrogen bond (O⋯N = 2.89 Å). In solution, comprehensive NMR experiments—including DMSO-d_6_ titration, variable-temperature spectroscopy, concentration-dependent chemical shift mapping, and NOE correlations—demonstrate that both proline-containing (**5**) and proline-free (**7** and **9**) NOPs adopt the same hydrogen-bonded turn in aprotic media. Strikingly, the motif remains intact in methanol-d_4_, a competitive protic solvent, attesting to the exceptional stability of the N-oxide constraint. DFT calculations reproduce the experimental geometries and energetic preferences, confirming that the N-oxide moiety alone is sufficient to nucleate the observed fold. Collectively, these findings redefine N-oxide as a versatile, residue-independent staple for programming peptide conformation, and expand the chemical space available for foldamer and peptidomimetic design.

## 2. Results and Discussion

### 2.1. Crystal Structure of NOP ***5***

Single crystals of NOP **5** were grown from CH_2_Cl_2_/n-hexane and analyzed by X-ray diffraction at 100 K. The asymmetric unit contains two symmetry-independent molecules that adopt nearly identical conformations. Each molecule folds into a six-membered ring stabilized by an intramolecular N–O⋯H–N hydrogen bond between the N-oxide oxygen and the adjacent amide NH (Figure 2). For the left molecular bond, the hydrogen bond distance (O--H) and angle (∠N-H--O) was 1.974 Å and 141.11°, respectively. For the right molecular bond, the hydrogen bond distance (O--H) and angle (∠N-H--O) was 1.991 Å and 131.13°, respectively. In addition to this, two intermolecular hydrogen bonds were observed between two molecules (the top one: O--H = 1.957Å, ∠N-H--O = 175.46°; the bottom one: O--H = 2.049 Å, ∠N-H--O = 176.75°). These metrics are consistent with medium-strong hydrogen bonds and provide the first crystallographic evidence that an N-oxidized peptide can nucleate a defined secondary structure independent of external hydrogen-bond partners.

### 2.2. Hydrogen-Bond Existence in Solution

To evaluate whether the N-oxide-driven fold persists outside the crystal lattice, we monitored amide proton chemical shift responses to solvent polarity, temperature and concentration under strictly anhydrous conditions.

#### 2.2.1. DMSO-d_6_ Titration Studies

To detect intramolecular hydrogen-bonding within the peptide backbone, we performed DMSO-d_6_ titrations following the protocol of Gellman and Miller [17]. For peptide **4** and NOP **5**, both containing a proline residue, NH_b_ exhibited large down-field shifts (Δδ 0.95 and 0.72 ppm, respectively; Figure 3A,B), indicating solvent exposure and the absence of protecting H-bonds. In contrast, NH_a_ showed only minor displacements (Δδ 0.15 ppm for **4**; 0.21 ppm for **5**), consistent with participation in a shielded, intramolecular interaction. Furthermore, the NH_a_ signal of NOP **5** is 2.2 ppm down-field of that in peptide **4** (10.22 vs. 8.05 ppm), reflecting the deshielding by the strongly electronegative N-oxide oxygen and confirming the formation of a six-membered N–O⋯H–N hydrogen bond.

On the other hand, we hypothesized that the proline unit in NOPs **2**, **3**, and **5** might be beneficial for the formation of intramolecular hydrogen bonds, and if the rigid structure of proline is necessary for the formation of intramolecular hydrogen bonds. In order to reveal the effect of the proline unit on the intramolecular hydrogen bond, we introduced the N-oxide into NOPs **7** and **9** without a proline structure for the first time, which was used in a conformational study compared with NOP **5**.

DMSO-*d*_6_ titration of peptide **6** and NOP **7** gave profiles superimposable on those of peptide **4** and NOP **5** (Figure 3C,D). Progressive addition of DMSO-*d*_6_ induced large shifts for NH_b_ (Δδ 1.05 ppm for **6**; 0.55 ppm for NOP **7**) but only minor changes for NH_a_ (Δδ 0.15 ppm for **6**; 0.39 ppm for NOP **7**), confirming solvent protection of the latter. The NH_a_ resonance of NOP **7** is 1.65 ppm down-field of that in peptide **6** (9.53 vs. 7.88 ppm), consistent with the deshielding by the N-oxide and formation of a six-membered N–O⋯H–N ring. Interestingly, in NOP **7**, the chemical shift variations in NH_a_ and NH_b_ are relatively close (ΔδNH_a_ = 0.39 ppm, ΔδNH_b_ = 0.55 ppm), showing a similar downfield trend unlike the other analogs. This feature likely originates from the greater conformational flexibility of the glycine residue, which—unlike proline—lacks a rigid pyrrolidine ring and allows rapid averaging between multiple backbone conformations in solution. As a result, the local magnetic environments of NH_a_ and NH_b_ become more alike. In addition, the electron-withdrawing phenyl group at the C-terminus may further deshield both amide protons to a comparable extent, reinforcing the observed similarity in their shifts.

The slightly larger Δδ(NH_a_) observed for NOP **7** (0.39 ppm) relative to NOP **5** (0.21 ppm) suggests that this C-terminal substituent weakens the intramolecular hydrogen bond through an inductive effect. To test this hypothesis, we replaced the phenyl group with a methyl ester to afford NOP **9**. Analogous DMSO-d_6_ titrations of peptide **8** and NOP **9** reproduced the same pattern: NH_b_ moved markedly (Δδ 1.01 and 0.81 ppm, respectively), whereas NH_a_ exhibited only small displacements (Δδ 0.10 and 0.23 ppm) and remained 1.4 ppm downfield in NOP **9** (Figure 3E,F). Thus, irrespective of the C-terminal substituent or the presence/absence of proline, the N-oxide consistently nucleates an intramolecular six-membered hydrogen bond, establishing this moiety—as opposed to proline—as the dominant conformational driver in these peptides.

#### 2.2.2. CD_3_OH Titration Studies

**Figure 4:**
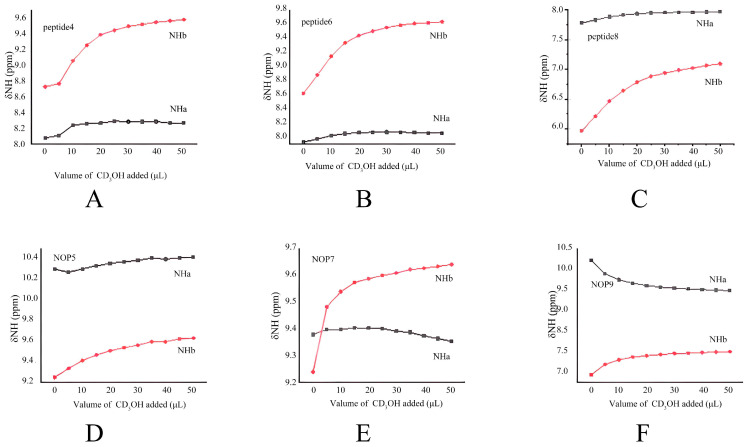
Amide proton chemical shifts plotted as a function of the amount of CD_3_OH added to 10 mM solutions of peptides **4**–**9** (**A**–**F**) in CDCl_3_ (0.5 mL) at room temperature.

To determine whether the N-oxide-stabilized hydrogen bond survives competition from a protic solvent, we subjected both control peptides and NOPs to CD_3_OH titration. The CD_3_OH titration results of peptides **4** and **6** and NOPs **5** and **7** were consistent with the results of the DMSO-*d_6_* addition studies (Figure 4A–D). The CD_3_OH titration results showed that the chemical shifts of NH_b_ change significantly in both peptides **4** and **6** (0.85 ppm and 1.02 ppm, respectively) and NOPs **5** and **7** (0.38 ppm and 0.40 ppm, respectively), suggesting that NH_b_ in all peptides was solvent-accessible. Meanwhile, all the NH_a_ underwent relatively small changes (∆δ_Nha_ < 0.21 ppm), revealing that the NH_a_ in all peptides was intramolecularly hydrogen bonded. In addition, the chemical shift changes of NH_a_ in both NOP **5** and NOP **7** (**5**: ∆δ_Nha_ = 0.12 ppm, **7**: ∆δ_Nha_ = 0.05 ppm) in the CD_3_OH titration experiment was significantly smaller than that in the DMSO-*d_6_* titration studies (**5**: ∆δ_Nha_ = 0.21 ppm, **7**: ∆δ_Nha_ = 0.39 ppm), which proved that the intramolecular hydrogen bond in NOPs **5** and **7** was even more stable in protic solvent than in the aprotic solvent.

The CD_3_OH titration study of peptide **8** was similar to that of peptide **6**. With increasing amounts of CD_3_OH, the chemical shifts of NH_b_ in peptide **8** showed large changes (**8**: ∆δ_NHb_ = 1.124 ppm) (Figure 4E), suggesting that NH_b_ was not involved in any intramolecular hydrogen bond. Meanwhile, the NH_a_ proton in peptide **8** was hydrogen-bonded, since it underwent relatively small chemical shift changes (**8**: ∆δ_Nha_ = 0.187 ppm). However, it was very interesting that the chemical shifts of NH_a_ and NH_b_ in NOP **9** both changed significantly (∆δ_Nha_ = −0.86 ppm, ∆δ_NHb_ = 0.644 ppm) in the CD_3_OH titration experiment; this indicated that the intramolecular hydrogen bonds in NOP **9** were easily affected by protic solvents. NH_a_ in NOP **9** was upshifted and the δ/ppm moved to high field with the increase of CD_3_OH addition (Figure 4F); this revealed that the hydrogen bond formed between NHa and N-oxide was very possibly dissociated by CD_3_OH to form other types of hydrogen bond. In the process of titrating protic solvent CD_3_OH, the difference in chemical shift change of NH_a_ in NOP **7** and NOP **9** may be caused by the difference in substituent at the terminal carbon.

#### 2.2.3. Concentration-Dependent Studies

**Figure 5:**
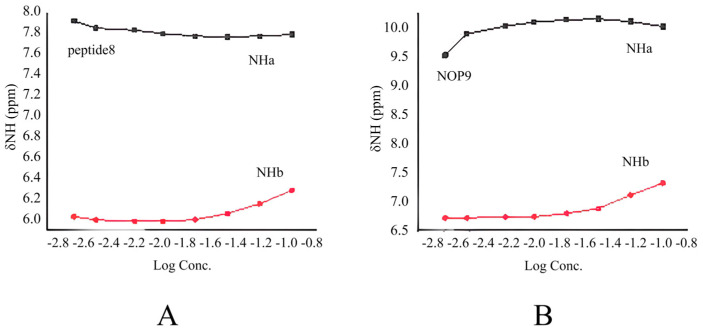
Amide proton chemical shifts plotted as a function of the logarithm of the concentration of peptide **8** (**A**) and NOP **9** (**B**) in CDCl_3_ at room temperature.

Due to the solubility of peptides **4–7** not reaching the requirement of the concentration-dependent experiment, peptide **8** and NOP **9** were chosen as the subjects of the concentration-dependent experiments. The spectroscopic data obtained upon concentrating peptide **8** and NOP **9** from 2 to 100 mM in CDCl_3_ displayed similar results to the DMSO-*d_6_* addition data. The relatively small chemical shift changes of NH_a_ in two peptides (**8**: ∆δ_NHa_ = 0.08 ppm; **9**: ∆δ_NHa_ = 0.13 ppm) (Figure 5A,B) implied clearly that NH_a_ is involved in an intramolecular hydrogen bond. However, the chemical shift of NH_b_ protons exhibited a relatively large change (**8**: ∆δ_NHb_ = 0.57 ppm; **9**: ∆δ_NHb_ = 0.60 ppm), which implied that NH_b_ might form intermolecular hydrogen bonds when the concentration of the peptide was increased.

#### 2.2.4. Variable Temperature Nuclear Magnetic Experiment

Analysis of the thermal behavior of hydrogen bonds in the peptides by NMR is an elegant technique to study the exact nature of intramolecular hydrogen bonds. In aprotic solvents such as DMSO-*d_6_*, when −Δδ/ΔT > 5 ppb K^−1^, the typical intramolecular hydrogen bonding was absent if the amide protons were solvent-exposed. When −Δδ/ΔT < 3ppb K^−1^, the amide proton was shielded from the solvent, due to hydrogen bonding with any electronegative group.

**Figure 6:**
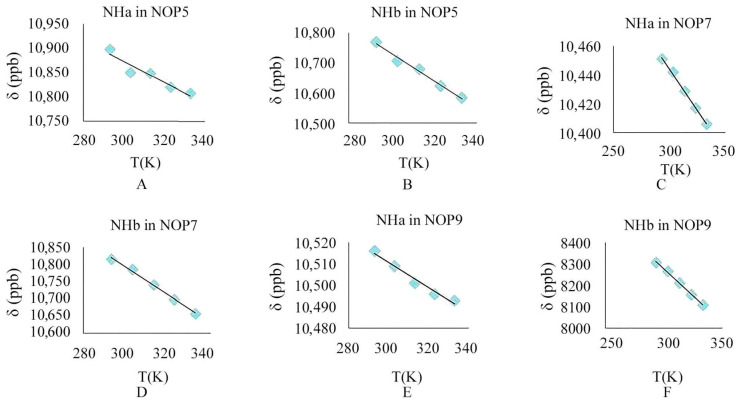
The hydrogen bond investigation with thermal coefficient plots for each amide proton of NOPs **5**, **7**, and **9** (Solvent: DMSO-*d*_6_, temperature: 293–333 K).

Further support for the intramolecular hydrogen bonding in NOPs was given from the temperature dependence of the amide NH resonances in DMSO-*d_6_*. The variable-temperature proton NMR spectroscopy results of NOPs are shown in Figure 5. Amide proton NH_a_ in NOP **5** showed a small variation with temperature (−Δδ_NHa_/ΔT = 2.13 ppb K^−1^) (Figure 6A), as expected for an intramolecularly H-bonded amide-NH group in polar solvent. As a comparison, the amide-NH_b_, which was incapable of intramolecularly H-bonding, showed a large upfield shift with increasing temperature (−Δδ_NHb_/ΔT = 4.56 ppb K^−1^) (Figure 6B). The chemical shift of NH_b_ in both NOPs **7** and **9** varies greatly with temperature (**7**: −Δδ_NHb_/ΔT = 4.04 ppb K^−1^; **9**: −Δδ_NHb_/ΔT = 5.04 ppb K^−1^) (Figure 6D,F), showing a strong temperature dependence, which indicated that NH_b_ did not form intramolecular hydrogen bonds in either NOP **7** or **9**. On the contrary, when the temperature rose from 293 K to 333 K, the chemical shifts of NH_a_ in NOPs **7** and **9** changed only 0.05 and 0.02 ppm, respectively. The change in temperature has little effect on the chemical shifts of NH_a_ (**7**: −Δδ_NHa_/ΔT = 1.15 ppb K^−1^; **9**: −Δδ_NHa_/ΔT = 0.59 ppb K^−1^) (Figure 6C,E), which fully confirmed that NH_a_ was involved in the intramolecular hydrogen bonds. The hydrogen bond strength has a positive correlation with the rate of chemical shift change. Compared with NOPs **7** and **9**, the −Δδ/ΔT plot of NOP **5** (Δδ/ΔT = 2.13 ppb K^−1^) was larger than that of either NOPs **7** (Δδ/ΔT = 1.15 ppb K^−1^) or **9** (Δδ/ΔT = 0.59 ppb K^−1^), indicating that the strength of the intramolecular hydrogen bond in NOP **5** formed by an NH_a_ and N-oxide dipole was stronger than that of NOPs **7** and **9**. Therefore, we could conclude that, although proline was not the main driving force for the formation of intramolecular hydrogen bonds, it can increase the strength of hydrogen bonds.

In NOP **7**, the NH_a_ and NH_b_ resonances overlap at δ ≈ 9.5 ppm in CDCl_3_ and exhibit identical DMSO-d_6_ titration slopes (Δδ 0.39 ppm for NH_a_ and 0.55 ppm for NH_b_). This convergence is ascribed to (i) the absence of the proline ring, which normally induces differential shielding on NH_b_, and (ii) the electron-withdrawing C-terminal phenyl group that deshields NH_b_, bringing its chemical shift close to that of NH_a_.

### 2.3. Nuclear Overhauser Effects (NOEs) of Dipeptides ***7*** and ***9***

**Figure 7:**
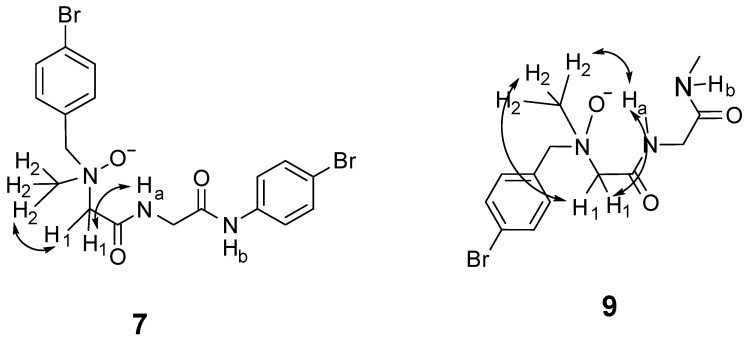
NOEs observed in a 50 mM solution of NOP **7** in DMSO-d_6_ and NOP **9** in CDCl_3_ at room temperature.

NOE experiments were recorded at 50 mM on NOP **7** in DMSO-d_6_ and on NOP **9** in CDCl_3_ (both 298 K). For NOP **7**, weak H_1_–NH_a_ and strong H_1_–H_2_ contacts were observed, whereas NOP **9** displayed medium H_1_–NH_a_ and strong H_2_–NH_a_/H_2_–H_1_ cross-peaks (Figure 7). The absence of longer-range enhancements indicates a single, well-defined conformer in each solvent. These through-space patterns mirror the six-membered N–O⋯H–N ring seen in the crystal structure of NOP **5**, confirming that the N-oxide enforces the same turn topology in both proline-containing and proline-free NOPs.

### 2.4. Computational Studies

**Figure 8:**
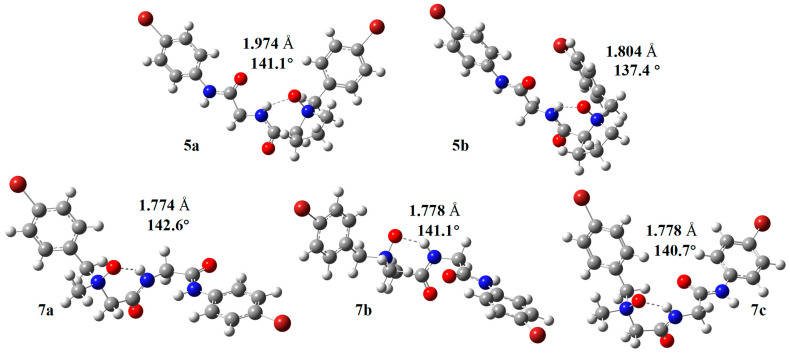
Calculated conformers and the relative energies of NOP **5** and NOP **7** in CHCl_3_. the N-H-O hydrogen bond length and angles are provided.

To gain additional understanding of the structure of the novel kind of conformation, we performed theoretical calculations on two kinds of NOP, including NOP **5** containing a proline unit and NOP **7** without a proline unit (Figure 8). The relative energies of the optimized conformers were analyzed at the B3LYP/6-31G(d,p) level in CHCl_3_ solution. Conformers lying within approximately 3 kcal mol^−1^ of the global minimum were regarded as thermally accessible under ambient conditions and therefore relevant to experimental observations, whereas those exceeding 5 kcal mol^−1^ were considered too unstable to make a measurable contribution. Attempts to optimize geometries initially arranged without the intramolecular N–O⋯H–N hydrogen bond either converged spontaneously to the hydrogen-bonded form or resulted in structures at least 5–8 kcal mol^−1^ higher in energy. These findings indicate that the six-membered hydrogen-bonded conformation represents the global minimum, while non-hydrogen-bonded arrangements are energetically disfavored and unlikely to exist in solution. This theoretical conclusion is consistent with the NMR data showing a protected NH_a_ proton and small temperature coefficients, confirming the robustness of the N–O⋯H–N interaction.

For NOP **5**, theoretical calculations were performed on its dimer crystal structures, which were properly stable both in gas and CHCl_3_ solution (Figure 8, **5a** and **5b**, Table S3) and could form six-membered-ring hydrogen bonds (**5a**: hydrogen bond length O--H = 1.974 Å, hydrogen bond angle ∠N-H--O: 141.1°; **5b**: hydrogen bond length O--H = 1.804 Å, hydrogen bond angle ∠N-H--O: 137.4°) with good structural parameters both in gas and solution. The conformation of **5a** was more similar to the crystal of NOP **5** than that of **5b**. Conformers **7a–c** (Figure 8, Table S3) displayed three stable conformations of NOP **7**. Conformer **7a** was the most stable, with a six-membered-ring hydrogen bond (hydrogen bond length O—H = 1.774 Å; hydrogen bond angle ∠N-H--O: 142.6°). Models **7b** and **7c** were both found to form a six-membered-ring hydrogen bond, but their single point energy was higher than that of **7a**, both in the gas phase and in CHCl_3_ solution. Theoretical calculation data showed the theoretical stable conformation of NOP **5** and NOP **7** corresponded to the above experimental results.

QTAIM analysis [24,25] on the optimized solution-phase conformers of NOPs **5** and **7** revealed bond critical points (BCPs) for the N–O⋯H–N hydrogen bonds (Figure S11). The electron densities ρ(r) and Laplacian values ∇^2^ρ(r) are consistent with medium-strength hydrogen bonding. QTAIM analysis on the stable conformers of **7** and **5** (solution phase) confirms medium-strength hydrogen bonding, with ρ(r) = 0.039–0.042 a.u., ∇^2^ρ(r) ≈ 0.13 a.u., and estimated E_HB_ values of 8.9 and 9.5 kcal mol^−1^ for NOPs **5** and **7**, respectively.

## 3. Materials and Methods

### 3.1. Chemistry

Experimental procedures and compound characterization data for the newly synthesized peptides (**4**–**9**, for ^1^H-NMR, ^13^C-NMR, and HRMS; see Figures S6–S35) are reported in the Supplementary Material. ^1^H- and ^13^C-NMR spectra were recorded either on an Agilent 400 MHz (400 and 100 for ^1^H and ^13^C, respectively), an Agilent 600 MHz (600 and 150 for ^1^H and ^13^C, respectively), a Bruker 600 MHz (600 and 150 for ^1^H and ^13^C, respectively), or a JEOL 600 MHz (600 and 150 for ^1^H and ^13^C, respectively) spectrometer at ambient temperature. The chemical shifts (δ) are reported in parts per million (ppm) relative to a trace amount of tetramethylsilane (0.00 ppm for ^1^H-NMR and ^13^C-NMR) from deuterated solvents (CDCl_3_, DMSO-*d_6_*). The coupling constants (*J*) are reported in hertz (Hz). High-resolution mass spectra (HRMS) were obtained on an Agilent 6540 accurate mass (Agilent Technologies, Santa Clara, CA, USA). Melting points were determined with an X-4A microscope (Shanghai Precision and Scientific Instrument Corporation, Shanghai, China) and were uncorrected. Optical rotations were measured on a Rudolph Autopol I (Rudolph Research Analytical, Hackettstown, NJ, USA).

### 3.2. X-Ray Diffraction

Single crystals of chemical formula C_20_H_21_Br_2_N_3_O_3_ were obtained. A suitable crystal was selected and measured on a SuperNova, Dual, Cu at zero, EosS2 diffractometer. The crystal was kept at 100 K during data collection. Using Olex20 [26], the structure was solved with Superflip [27,28]. The structure solution program used Charge Flipping, refined with ShelXL [29]. Refinement package used Least Squares minimization.

### 3.3. Computational Methods

Theoretical calculations were performed on NOPs **5** and **7** using the Gaussian 98 software package [5]. Geometry optimizations were conducted at the B3LYP/6-31G(d,p) level using the polarizable continuum model (PCM) for chloroform (CHCl_3_) [30]. Harmonic vibrational frequency calculations were performed on all optimized structures to confirm they correspond to true minima (no imaginary frequencies). To explore the conformational landscape, non-hydrogen-bonded starting geometries were prepared by setting H⋯O distances > 3.5 Å and rotating relevant backbone dihedrals to disfavor six-membered ring closure; these were likewise optimized under SDM(CHCl_3_).

Single-point energy evaluations were subsequently performed at the MP2/6-311G(d,p) level on the solution-phase optimized geometries to obtain more accurate relative energies. To quantify the hydrogen bond energies, QTAIM topological analysis was performed using the Multiwfn suite. High-quality wavefunctions for the optimized conformers were generated via single-point calculations at the LC-wPBE/6-311+G(d,p) level with the solvation model density (SDM) approach in CHCl_3_ [31]. Bond critical points (BCPs) corresponding to the N–O⋯H–N interactions were identified, and electron densities ρ(r) and Laplacian values ∇^2^ρ(r) were extracted. Hydrogen bond energies (E_HB_) were estimated using Espinosa’s formula as implemented in Multiwfn.

## 4. Conclusions

In conclusion, we have synthesized and characterized three novel N-oxidized peptides (NOPs **5**, **7**, and **9**) that consistently adopt a six-membered intramolecular hydrogen bond between the N-oxide oxygen and an adjacent amide proton. This structural motif was first confirmed by X-ray crystallography in the proline-containing NOP **5**, and subsequently validated in glycine-based analogs (**7** and **9**) through comprehensive NMR studies in both aprotic and protic solvents. Notably, the hydrogen bond persists in methanolic solution, underscoring its stability even under competitive hydrogen-bonding conditions. DFT calculations corroborate the experimental observations and highlight the energetic favorability of the N-oxide-induced conformation. These findings establish N-oxide as a robust and generalizable constraint for peptide backbone preorganization, independent of proline. This strategy opens new avenues for designing peptidomimetics with enhanced conformational rigidity and solvent-resistant secondary structures, complementing recent theoretical insights into N-oxide hydrogen bonding behavior under microsolvation conditions.

## Data Availability

The original contributions presented in this study are included in the article/Supplementary Material. Further inquiries can be directed to the corresponding author.

## Supplementary Materials

The following supporting information can be downloaded at: https://www.mdpi.com/article/10.3390/molecules30244676/s1, Experimental procedures and characterization data for intermediates **1s**–**6s** and peptides **4**–**9**, Page 2–7; DMSO-*d_6_* addition studies, Page 8; CD_3_OH titration studies, Page 9; Concentration dependent NMR studies Page 10; Variable-temperature NMR studies Page 11,12; NOEs for g-NOP **7** and **9**, Page 13,14; X-ray diffraction of p-NOP **5**, Page 15; Thermodynamic calculations, Page 15–20; NMR and HRMS spectra for intermediates **1s**–**6s** and peptides **4**–**9**, Page S21.
