# Supplementary material for: Proline-Free Local Turn via N-Oxidation: Crystallographic and Solution Evidence for a Six-Membered N–O⋯H–N Ring
Source: Molecules. 2025 Dec 5;30(24):4676. doi: 10.3390/molecules30244676 (PMC12736079; doi:10.3390/molecules30244676)
Supplement: Supplementary file 1 [file molecules-30-04676-s001.zip › molecules-3945023-supplementary.pdf]

Supplementary Material

Content

Experimental procedures and characterization data for intermediates **1s-6s** and peptide**s 4-9** S2

DMSO-*d_6* addition studies S7

CD_3OH titration studies S8

Concentration dependent NMR studies S9

Variable-temperature NMR studies S10

NOEs for g-NOP **7** and **9** S13

X-ray diffraction of p-NOP **5** S14

Thermodynamic calculations S15

NMR and HRMS spectra for intermediates **1s-6s** and peptide**s 4-9** S21

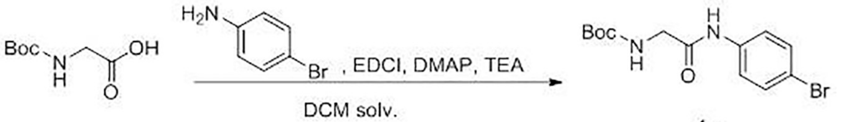

1s

tert-butyl (2-((4-bromophenyl)amino)-2-oxoethyl)carbamate **1s**: A mixture of Boc-glycine (600 mg, 3T.43 mmol), 1-ethyl-3-(3-dimethylaminopropyl) carbo-diimidehydrochloride (788 mg, 4.11 mmol) (EDCI), 4-dimethylaminopyridine (126 mg, 1.03 mmol) (DMAP), triethylamine (1.2 mL, 8.58 mmol) and 4-bromoaniline (648 mg, 3.77 mmol) in dry dichloromethane (50 mL) (DCM) was stirred at room temperature for 12 hours. The reaction was monitored by TLC. On completion of the reaction, it was washed with water (2x30 mL), brine (1x20 mL). The organic layer was dried over anhydrous sodium sulfate and was concentrated under reduced pressure to give the crude product which was purified by column chromatography on silica gel, employing acetone and petroleum ether as eluent to yield desired product **1s** as white solid (730 mg, Yield: 65%); m.p 163.7~167.5 ℃; ^{1}HNMR (400MHz, $CDCl_3$) δ 8.35 (s, 1H), 7.41 (s, 4H), 5.3 (s, 1H), 3.92 (d, J = 5.6 Hz, 2H), 1.47(s, 9H); ^{13}C NMR (101 MHz, DMSO-d_6) δ 168.86, 156.35, 138.73, 131.95, 121.40, 115.12, 78.50, 44.24, 28.62.

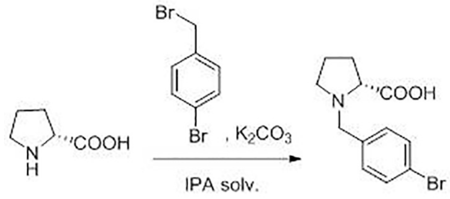

2s

(4-bromobenzyl)-L-proline **2s**: A mixture of (L)-proline (500 mg, 4.35 mmol) and K_2CO_3 (1790 mg, 13.04 mmol) in isopropanol (60 mL) was treated with 4-bromobenzyl bromide (1142 mg, 4.57 mmol). The reaction mixture was stirred at 40 ℃ for 6 h. The reaction mixture was cooled to room temperature and the pH was adjusted to 4 with concentrated HCl. Then most of isopropanol was evaporated and water was added to the residual. The mixture was extracted with ethyl acetate(EA). The organic layers were combined and concentrated to give a light yellow solid which was washed with EA to yield **2s** as pure white solid (790 mg, Yield: 64.4%); m.p. 216.0~219.1 ℃; $[\alpha]_D^{29.3}$ = -14.3 (c=0.105, MeCN); ^{1}H NMR (400 MHz, DMSO-d_6) δ 7.58 (d, J = 8.1 Hz, 2H), 7.47 (d, J = 7.6 Hz, 2H), 4.35 (d, J = 12.7 Hz, 1H), 4.17 (d, J = 12.5 Hz, 1H), 4.06~3.95 (m, 1H), 3.31 (m, 1H), 3.02 (m, 1H), 2.31 (m, 1H), 2.00~1.74 (m, 3H); ^{13}C NMR (101 MHz, DMSO-d_6) δ 171.0, 133.1, 131.9, 122.8, 65.9, 56.7, 54.2, 28.7, 22.7; HRMS (ESI-TOF) m/z [M+H] cald for $C_{12}H_{14}BrNO_2$ 284.0286 , found 284.0285.

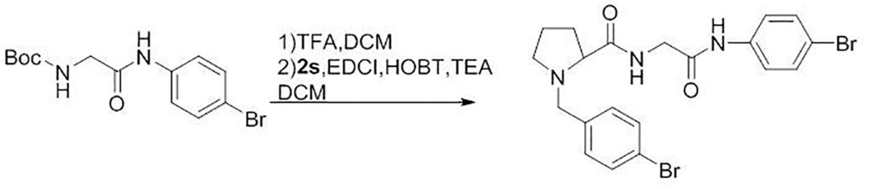

1s 4

(S)-1-(4-bromobenzyl)-N-(2-((4-bromophenyl)amino)-2-oxoethyl)pyrrolidine-2-carboxamide **4**: **1s** (200 mg, 0.6 mol.) was dissolved in solution of CH_2Cl_2 (10 mL) and trifluoroacetic acid (3 mL). After

the solution had been kept at room temperature for 60 min, it was concentrated to dryness, then add the 1-ethyl-3-(3-dimethylaminopropyl) carbodiimideh-ydrochloride (149 mg, 0.66 mmol) (EDCI), 1-hydroxybenzotriazole (95 mg, 0.66 mmol) (HOBT), triethylamine (0.34 mL, 1.8 mmol), dry product **2s**, dichloromethane (DCM) (40 mL) and N,N-dimethylformamide (6 mL) (DMF), the mixture was stirred at room temperature for 12 hours. The reaction was monitored using TLC. On completion of the reaction, the mixed solution was concentrated and washed with water (30 mL), brine (1x20 mL), dried over anhydrous sodium sulfate and the solvent was evaporated under reduced pressure to give the crude product which was purified by column chromatography using silica gel, employing methyl alcohol and DCM as eluent to yield desired product **4** as white solid (125 mg, Yield: 50%); m.p. 220~223.3 ℃; $[\alpha]_D^{29.3}$ = -26.0 (c=0.100, MeCN); ^{1}HNMR (400MHz, $CDCl_3$) δ 8.69 (s, 1H), 8.06 (s, 1H), 7.43 (s, 6H), 7.20 (d, *J* = 7.9 Hz, 2H), 4.00 (s, 2H), 3.79 (d, *J* = 12.9 Hz, 1H), 3.55 (d, *J* = 12.9 Hz, 1H), 3.26 (dd, *J* = 10.1, 4.3 Hz, 1H), 3.07 (t, *J* = 7.1 Hz, 1H), 2.40 (q, *J* = 9.5 Hz, 1H), 2.26 (dt, *J* = 19.7, 9.7 Hz, 1H), 1.90 (m, *J* = 8.4 Hz, 1H), 1.77 (m, *J* = 18.4, 7.8 Hz, 2H); ^{13}C NMR (101 MHz, $CDCl_3$) δ 174.2, 169.3, 138.7, 132.0, 131.6, 131.4, 121.4, 120.4, 115.2, 67.4, 58.3, 53.3, 42.7, 30.2, 23.7; HRMS (ESI-TOF) m/z [M+H] cald for $C_{20}H_{22}Br_2N_3O_2$ 494.0079, found 494.0092.

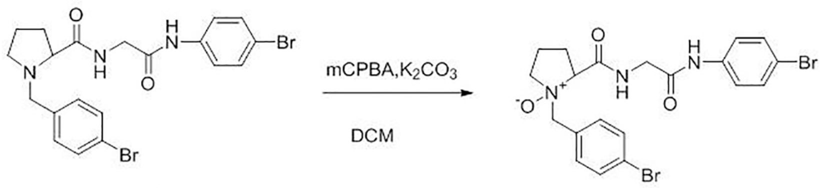

4 5

(2S)-1-(4-bromobenzyl)-N-(2-((4-bromophenyl)amino)-2-oxoethyl)-1-(l1-oxidanyl)-1l4-pyrrolidine-2-carboxamide **5:** A stirred solution of peptidomimetic **4** (100 mg, 0.12 mmol) in CH_2Cl_2 (15 mL) was treated with solid K_2CO_3 (4 mol equiv) and 85% mCPBA (49 mg, 0.29 mol). The resulting mixture was stirred at -78 ℃ temperature for 7 h, at which time complete comsumption of starting material was observed by TLC. When reaction liquid rised to room temperature, the solid was separated by filtration, and the filtrate was dried over anhydrous sodium sulfate and concentrated under reduced pressure, the crude product which was purified by column chromatography using silica gel, employing methyl alcohol and DCM as eluent to yield desired product **5** as white solid (44 mg, Yield: 87%); m.p. 162.7~168.5 ℃; $[\alpha]_D^{29.3}$ = -7.1 (c=0.070, MeCN); ^{1}HNMR (400MHz, $CDCl_3$) δ 10.65 (s, 1H), 10.55 (s, 1H), 7.59 (dd, *J* = 8.7 Hz, 4H), 7.48 (dd, *J* = 19.2, 8.5 Hz, 4H), 4.6 (dd, 2H), 4.02 (m, *J* = 17.0, 7.7 Hz, 2H), 3.91 (ddd, *J* = 5.3 Hz, 1H), 3.49 (dd, *J* = 10.2 Hz, 1H), 3.02 (m, 1H), 2.25 (q, *J* = 8.1 Hz, 2H), 2.06 (m, *J* = 7.3 Hz, 1H), 1.89 (m, 1H); ^{13}C NMR (101 MHz, $CDCl_3$) δ 168.2, 168.1, 138.8, 134.9, 131.9, 131.4, 131.0, 123.3, 121.6, 115.3, 75.1, 68.1, 66.3, 43.1, 26.0, 20.4; HRMS (ESI-TOF) m/z [M+H] cald for $C_{20}H_{22}Br_2N_3O_3$ 510.0028, found 510.0046.

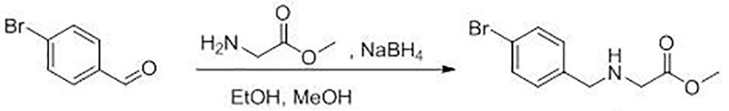

3s

methyl (4-bromobenzyl)glycinate **3s**: 4-bromobenzaldehyde (500 mg, 2.7 mmol) and glycine methyl ester hydrochloride (340 mg, 2.7 mmol) were dissolved in ethanol (7.5 ml) under nitrogen with stirring and triethylamine (0.73 ml, 5.2 mmol) was added. The reaction mixture was heated to 50℃ for 1 hour,

then evaporated to dryness and the residue taken up in methanol (7.5 ml) and cooled in an ice bath. Sodium borohydride (123 mg, 3.24 mmol) was added portionwise and stirring continued until no starting material remained as determined by TLC. The reaction mixture was concentrated to dryness and partitioned between DCM and water and the phases separated. The organic layer was dried over sodium sulphate, filtered and concentrated before purification on silica eluting with ethyl acetate(EA) and petroleum ether to give desired product **3s** as yellow oil. (440 mg, Yield: 63%.); ^{1}HNMR (400MHz, $CDCl_3$) δ 7.31 (d, J = 8.4 Hz, 2H), 7.09 (d, J = 8.4 Hz, 2H), 3.63 (s, 2H), 3.60 (s, 3H), 3.27 (s, 2H), 2.07 (s, 1H). ^{13}C NMR (101 MHz, $CDCl_3$) δ 172.69, 138.47, 131.44, 129.88, 120.88, 52.45, 51.74, 49.72.

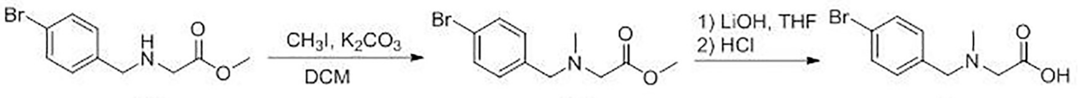

methyl N-(4-bromobenzyl)-N-methylglycinate **4s** : A stirred solution of product **3s** (935 mg, 3.64 mmol) and K_2CO_3(754 mg, 5.46 mol) in CH_2Cl_2 (15 mL) was added Methyl iodide (0.19 ml, 2.91 mmol) and cooled in an ice bath. Then the resulting mixture was stirred at room temperature for 14 h. The solid was filtered and washed with DCM (2×5 mL). The filtrate was dried over Na_2SO_4 and concentrated before purification on silica eluting with ethyl acetate (EA) and petroleum ether to give desired product **4s** as yellow oil (488 mg, Yield: 50%.); ^{1}HNMR (400MHz, $CDCl_3$): δ 7.40 (d, J = 8.4 Hz, 2H), 7.19 (d, J = 8.4 Hz, 2H), 3.67 (s, 3H), 3.59 (s, 2H), 3.23 (s, 2H), 2.33 (s, 3H); ^{13}C NMR (101 MHz, $CDCl_3$): δ 172.69, 138.47, 131.44, 129.88, 120.88, 52.45, 51.74, 49.72.

N-(4-bromobenzyl)-N-methylglycine **5s**: To a solution of **4s** (488 mg, 1.8 mmol) in THF (10 mL) and H_2O (2 mL) was added $LiOH{\cdot}H_2O$ (83.1 mg, 1.98 mmol). The mixture was stirred room tempreture for 3 h, then the organic solvent was evaporated under reduced pressure. The aqueous layer was adjusted to 7 with 0.5N HCl, and concentrated to dryness. No further purification is required; HRMS (ESI-TOF) m/z [M+H] cald for $C_{10}H_{13}BrNO_2$ 258.0130, found 258.0128.

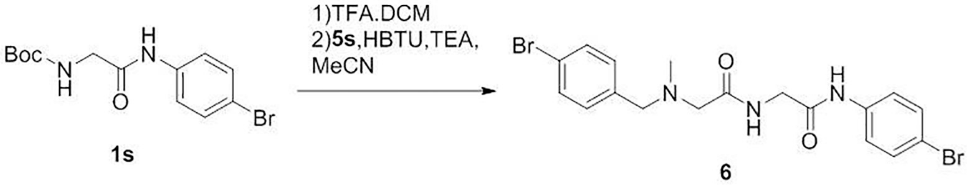

2-((4-bromobenzyl)(methyl)amino)-N-(2-((4-bromophenyl)amino)-2-oxoethyl)acetamide **6**: **1s** (600 mg, 1.83 mol) was dissolved in mixture of CH_2Cl_2 (6 mL) and trifluoroacetic acid (1.5 ml). After the solution had been kept at room temperature for 60 min., it was concentrated to dryness, then add the O-Benzotriazole-N,N,N',N'-tetraMethyl-uroniuM-hexafluorophosphate (831 mg, 2.19 mmol) (HBTU), triethylamine (0.76 mL, 5.49 mmol), dry product **5s** (470 mg, 1.83 mmol), dry acetonitrile (20 mL) (MeCN), the mixture was stirred at roomtemperature for 12 hours. The reaction was monitored using TLC. On completion of the reaction, the mixed solution was concentrated and washed with water (30 mL), brine (2x30 mL), dried over anhydrous sodium sulfate and the solvent was evaporated under reduced pressure to give the crude product which was purified by column chromatography using silica gel, employing methyl alcohol and DCM, ether as eluent to yield desired product **6** as white solid (393 mg, Yield: 46%); m.p. 156.6~160.8 ℃; ^{1}HNMR (400MHz, DMSO-d_6) δ 10.12 (s, 1H), 8.07 (s, 1H),

7.5 (m, 6H), 7.35 (d, J = 7.8 Hz, 2H), 3.92 (d, J = 5.3 Hz, 2H), 3.54 (s, 2H), 3.54 (s, 2H), 2.18 (s, 3H); ^{13}C NMR(101 MHz, DMSO-d_6) 170.6, 168.3, 138.7, 138.3, 132.0, 131.5, 135.4 , 121.4, 120.6, 115.2, 60.9, 60.8, 60.9, 42.6. HRMS (ESI-TOF) m/z [M+H] cald for $C_{18}H_{19}Br_2N_3O_2$ 467.9922, found 467.9974.

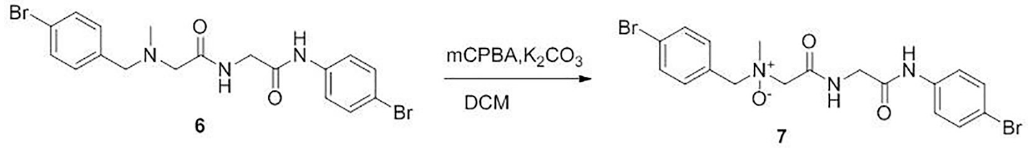

2-((4-bromobenzyl)(methyl)(l1-oxidanyl)-l4-azanyl)-N-(2-((4-bromophenyl)amino)-2-oxoethyl) Acetamide **7**: To a stirred solution of peptidomimetic **6** (220 mg, 0.43 mmol) in CH_2Cl_2 (15 mL) was added solid K_2CO_3 (119 mg, 0.86 mmol) and 85% m-CPBA (106 mg, 0.56 mol). The resulting mixture was stirred at -78 ℃ temperature for 7 h, at which time complete comsumption of starting material was observed by TLC. When reaction liquid rised to room temperature, the solid was separated by filtration, and the filtrate was dried over anhydrous sodium sulfate and concentrated under reduced pressure, the crude product which was purified by column chromatography using silica gel, employing methyl alcohol and DCM, ether as eluent to yield desired product **7** as white solid (190 mg, Yield: 91%); ^{1}H NMR (400 MHz, DMSO-d_6) δ 10.79 (s, 1H), 10.44 (t, J = 6.1 Hz, 1H), 7.69~7.38 (m, 8H), 4.50 (s, 2H), 3.96 (d, J = 5.6 Hz, 2H), 3.81 (q, J = 10.6 Hz, 2H), 3.01 (s, 3H); ^{13}C NMR (101 MHz, DMSO-d_6) 168.38, 166.13, 138.86, 135.23, 131.84, 131.41, 130.82, 123.33, 121.71, 115.25, 71.91, 68.58, 56.37, 43.34; HRMS (ESI-TOF) m/z [M+H] cald for $C_{18}H_{19}Br_2N_3O_3$ 483.9871, found 483.9863.

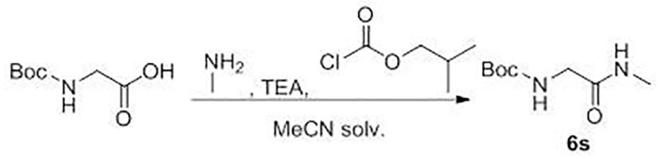

tert-butyl (2-(methylamino)-2-oxoethyl)carbamate **6s**: Isobutyl chloroformate (0.92 mL, 6.3 mmol) was successively added to a solution of Boc-Gly(1 g, 5.7 mmol) and TEA(0.88 mL, 6.3 mmol) in DCM (20 mL) at -20 ℃. After an activation period of 30 min, 40 % aqueous methylamine (15 mL, 12 mmoL) was added to the solution, and the resulting solution was stirred for 2 h at -20 ℃ prior to the addition of 5% $NaHCO_3$ (30 mL). After 30 min at room temperature, the aqueous phase was extracted with CH_2Cl_2 (3×100 mL). The combined organic layer was washed with 5 % $NaHCO_3$ (2×30 mL) and dried (Na_2SO_4). The solvent was evaporated under reduced pressure to give the crude product which was purified by column chromatography using silica gel, methyl alcohol and DCM, ether as eluent to yield desired product **6s** as white solid (800 mg, Yield: 81%); m.p. 53.7~53.9 ℃; ^{1}H NMR (400 MHz, $CDCl_3$) δ 6.46 (s, 1H), 5.36 (m,1H), 3.76 (d, J = 4.9 Hz, 2H), 2.79 (m, 3H), 1.42 (s, 9H); ^{13}C NMR (101 MHz, $CDCl_3$) δ 170.09, 156.11, 80.24, 44.27, 28.28, 26.11.

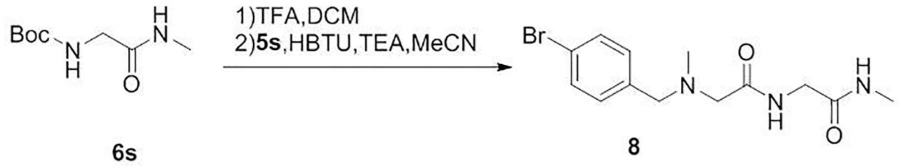

2-((4-bromobenzyl)(methyl)amino)-N-(2-(methylamino)-2-oxoethyl)acetamide **8**: **6s** (400 mg, 2.13 mmol) was dissolved in mixture of CH_2Cl_2 (8 mL) and trifluoroacetic acid (2 ml). After the solution had been kept at room temperature for 60 min., it was concentrated to dryness, then add the O-Benzotriazole-N,N,N',N'-tetraMethyl-uroniuM-hexafluorophosphate (970 mg, 2.56 mmol) (HBTU), triethylamine (0.89 mL, 6.39 mmol), dry product **5s** (547 mg, 2.13 mmol), dry acetonitrile (20 mL) (MeCN), the mixture was stirred at roomtemperature for 8 hours. The reaction was monitored using TLC. On completion of the reaction, the mixed solution was concentrated and washed with water (30 mL), brine (2x30 mL), dried over anhydrous sodium sulfate and the solvent was evaporated under reduced pressure to give the crude product which was purified by column chromatography using silica gel, employing methyl alcohol and DCM, ether as eluent to yield desired product **8** as white solid (336 mg, Yield: 46%); m.p. 136.0~144.1 ℃; ^{1}H NMR (400 MHz, $CDCl_3$) δ 7.80 (t, J = 5.7 Hz, 1H), 7.44 (d, J = 8.1 Hz, 2H), 7.20 (d, J = 8.1 Hz, 2H), 6.45 (s, H), 3.91 (d, J = 5.5 Hz, 2H), 3.53 (s, 2H), 3.05 (s, 2H), 2.79 (d, J = 4.8 Hz, 3H), 2.28 (s, 3H).^{13}C NMR (101 MHz, $CDCl_3$): 171.36, 169.27, 136.70, 131.63, 130.66, 121.41, 61.73, 60.28, 43.29, 42.71, 26.21; HRMS (ESI-TOF) m/z [M+H] cald for 328.0661, found 328.0659.

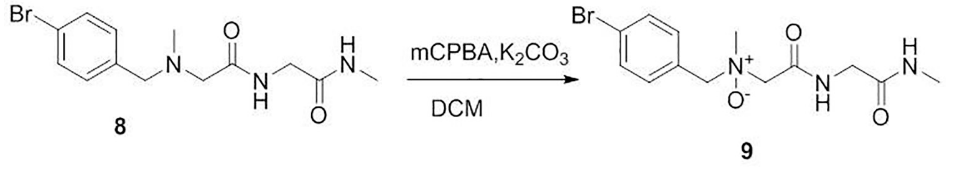

2-((4-bromobenzyl)(methyl)(l1-oxidanyl)-l4-azanyl)-N-(2-(methylamino)-2-oxoethyl)acetamide **9:** To a stirred solution of peptidomimetic **8** (170 mg, 0.5 mmol) in CH_2Cl_2 (15 mL) was added solid K_2CO_3 (139 mg, 1 mmol) and 85% m-CPBA (130 mg, 0.65 mol). The resulting mixture was stirred at -78℃ temperature for 5 h, at which time complete comsumption of starting material was observed by TLC. When reaction liquid rose to room temperature, the solid was separated by filtration, and the filtrate was dried over anhydrous sodium sulfate and concentrated under reduced pressure, the crude product which was purified by column chromatography using silica gel, employing methyl alcohol and DCM, ether as eluent to yield desired product **9** as white solid (150 mg, Yield: 84%).^{1}H NMR (400 MHz, $CDCl_3$) δ 10.14 (t, J = 6.5 Hz, 1H), 7.57 (d, J = 8.4 Hz,2H), 7.42 (d, J = 8.5 Hz, 2H), 4.59 (d, J = 12.7 Hz, 1H), 4.42 (d, J = 12.7 Hz, 1H), 4.04 (dd, J = 16.5, 6.5 Hz, 1H), 3.93 (dd, J = 16.4, 6.1 Hz, 1H), 3.86(d, J = 13.6 Hz, 1H), 3.74 (d, J = 13.5 Hz, 1H), 3.17 (s, 3H), 2.79 (d, J = 4.7 Hz, 3H) ; ^{13}C NMR (101 MHz, $CDCl_3$) :169.30, 165.29, 134.07, 132.13, 128.46, 124.81, 73.78, 67.66, 56.62, 42.92, 26.19; HRMS (ESI-TOF) m/z [M+H] cald for 344.0610, found 344.0623.

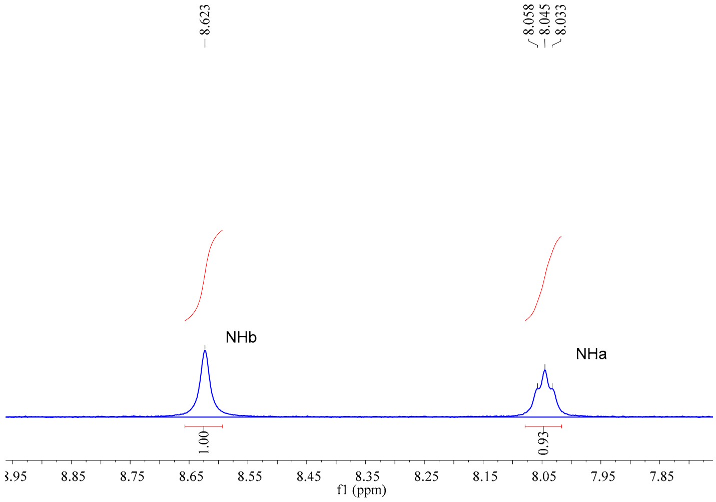

Supplementary Figure S1. Locally amplified spectra of peptide **4**.

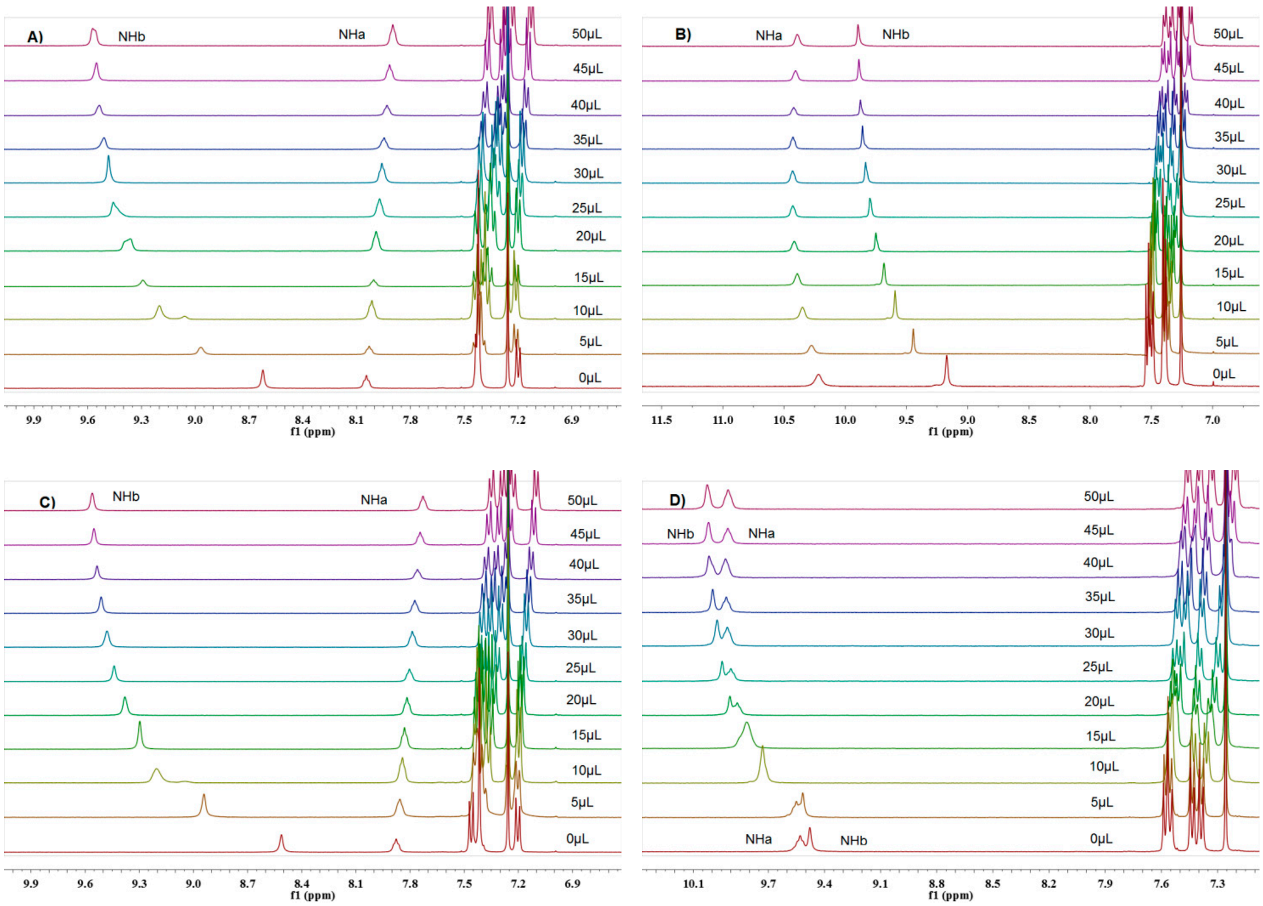

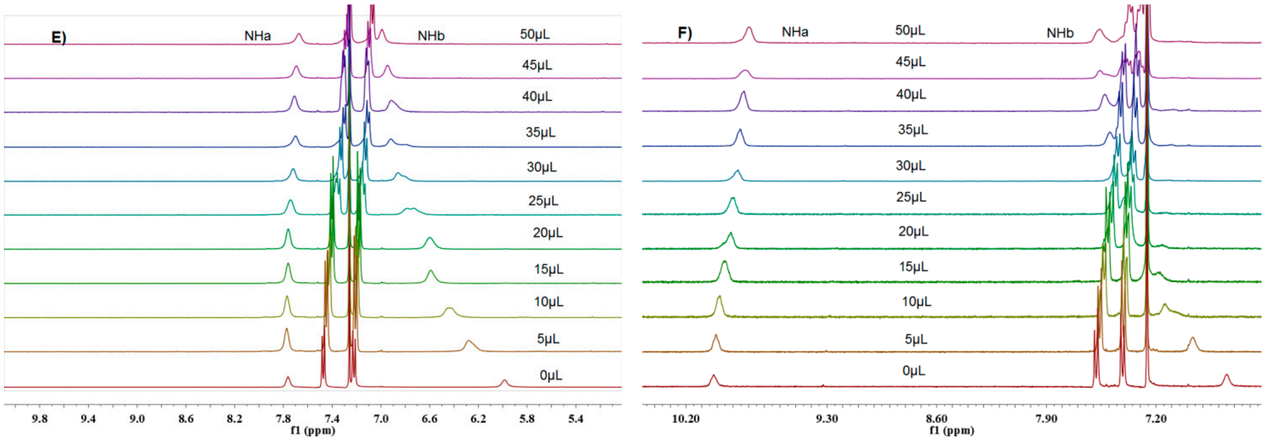

Supplementary Figure S2. ^{1}H NMR chemical shifts of amide protons of compounds **4-9** (5mM in $CDCl_3$) (A-F) when increasing amount of DMSO-d_6 was added at room temperature.

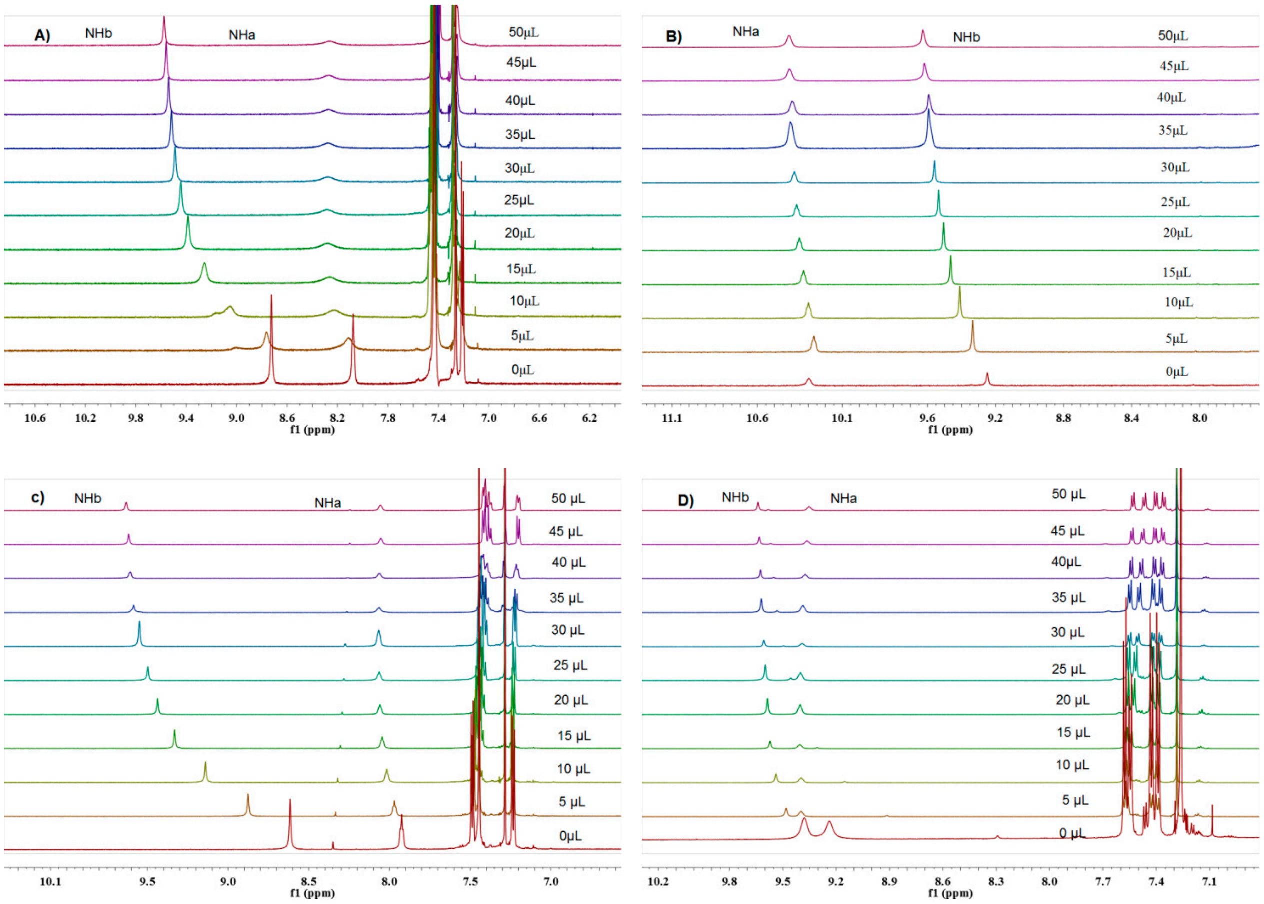

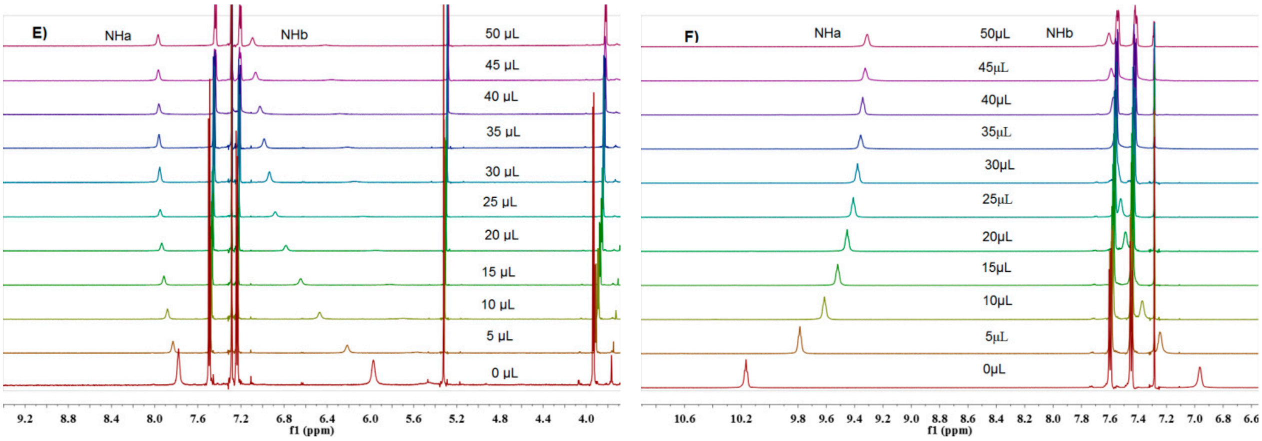

Supplementary Figure S3. ^{1}H NMR chemical shifts of amide protons of compounds **4-9** (5mM in $CDCl_3$) (A-F) when increasing amount of CD_3OH was added at room temperature.

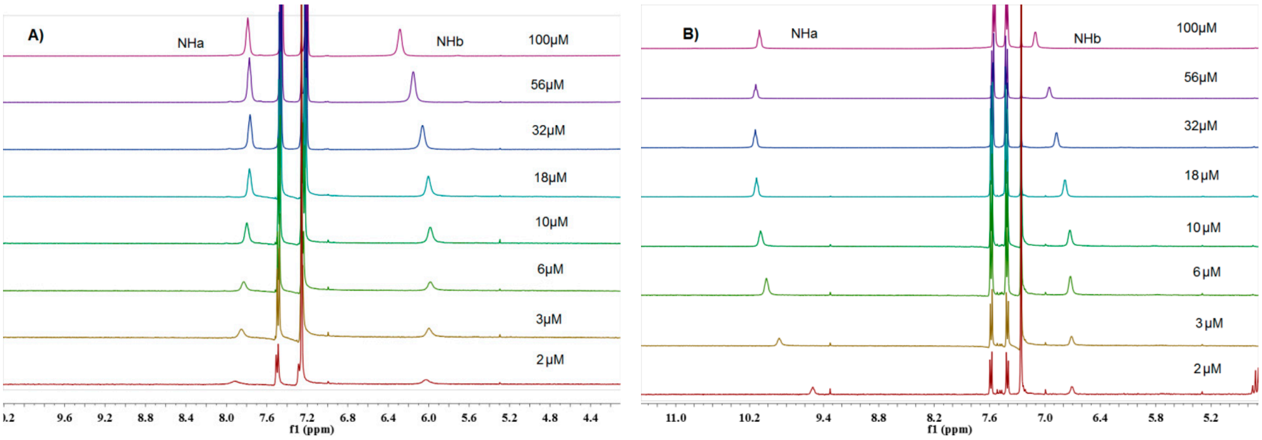

Supplementary Figure S4. Amide proton chemical shifts plotted as a function of the logarithm of the concentration of peptides **8** (A) and g-NOP **9** (B) in $CDCl_3$ at room temperature.

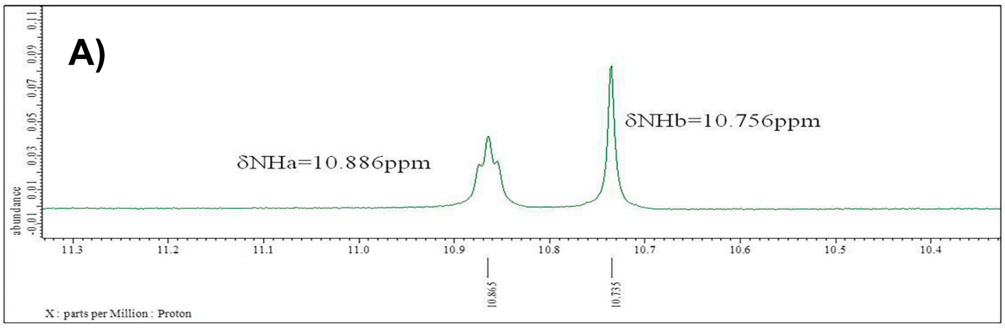

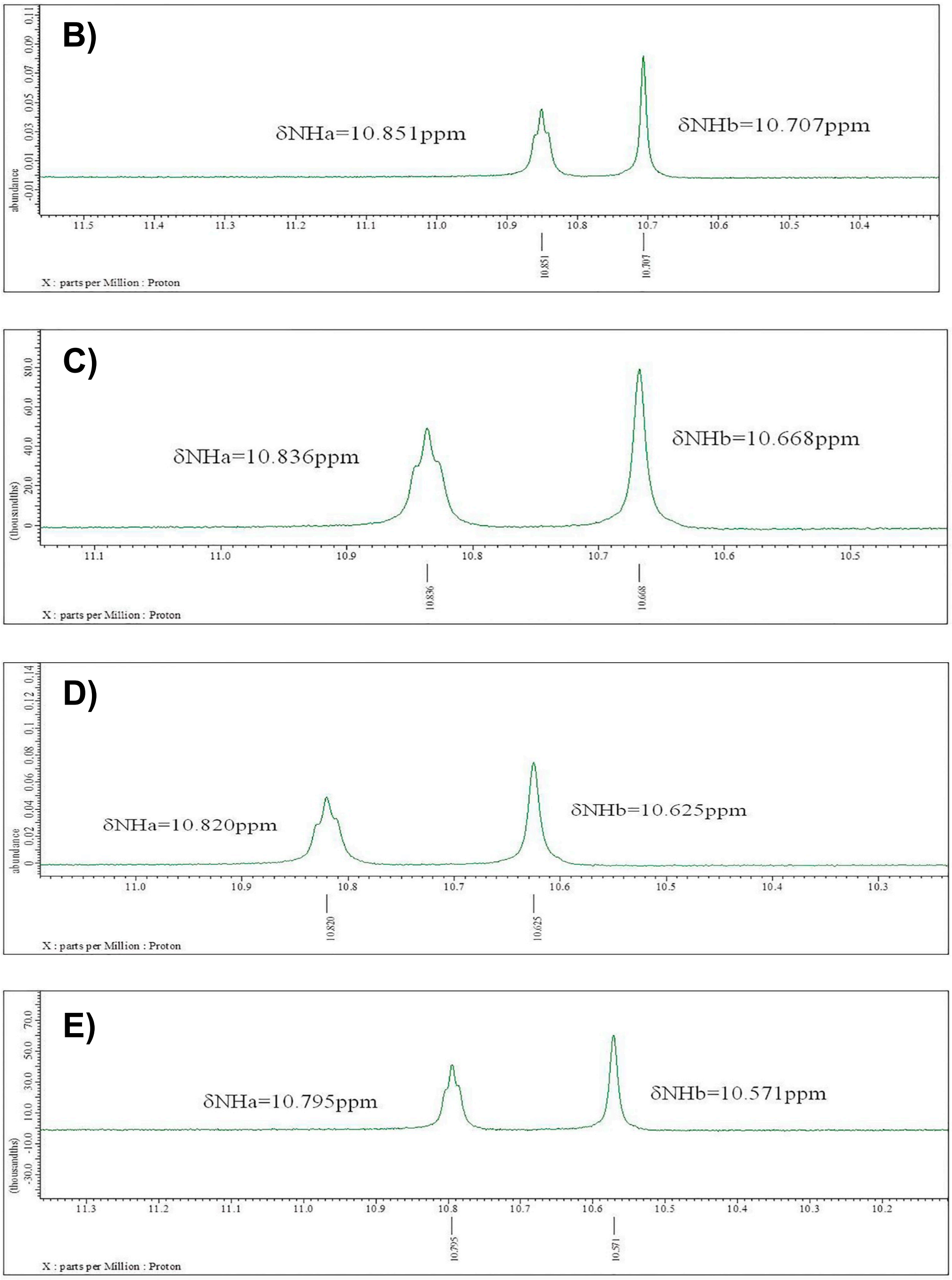

Supplementary Figure S5. The chemical shifts of amide protons in p-NOP **5** at 293K (A), 303K (B), 313K (C), 323K (D), and 333K (E).

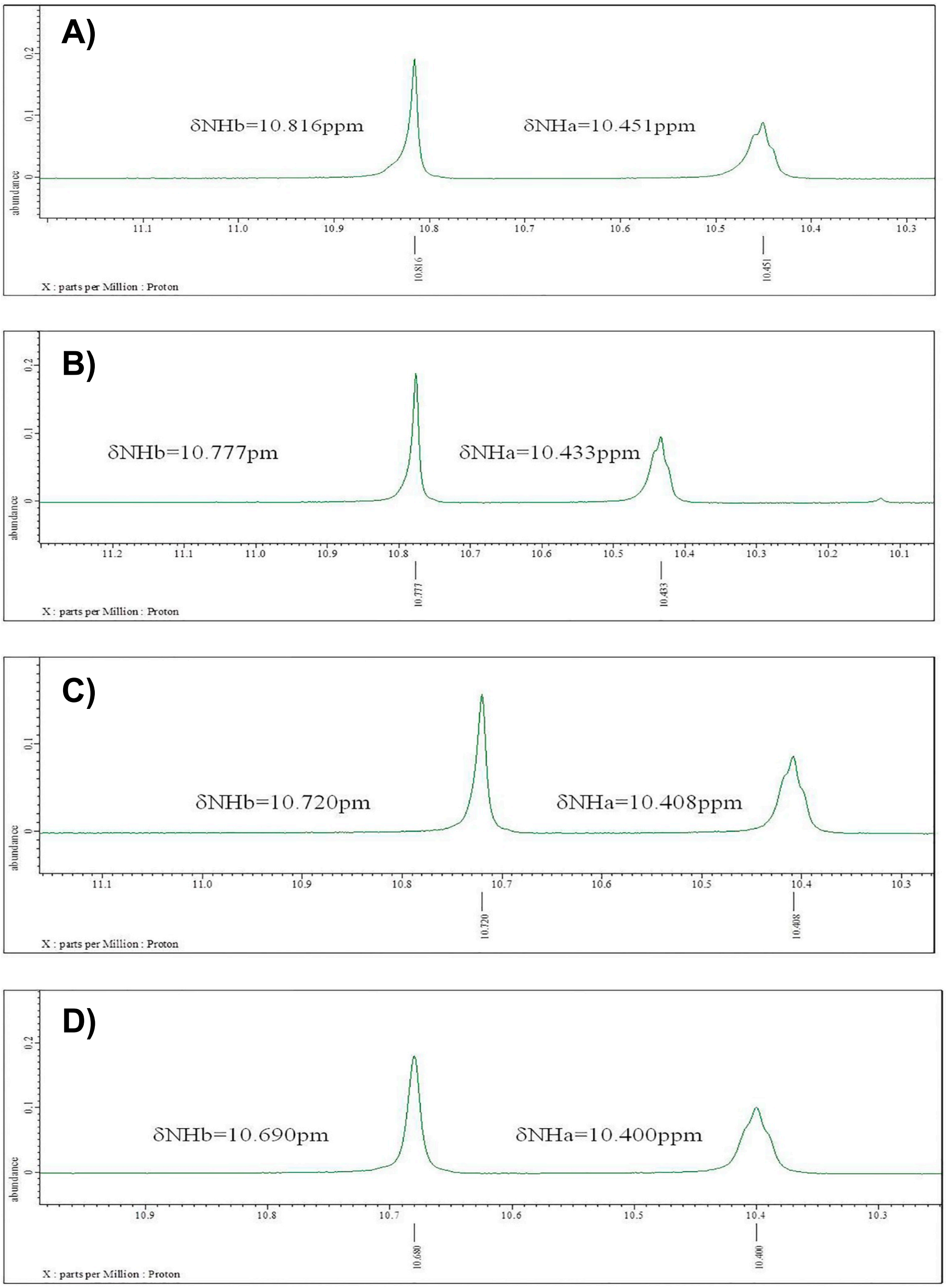
A)
δNHb=10.816ppm
δNHa=10.451ppm
10.816
10.451
X : parts per Million : Proton
B)
δNHb=10.777pm
δNHa=10.433ppm
10.777
10.433
X : parts per Million : Proton
C)
δNHb=10.720pm
δNHa=10.408ppm
10.720
10.408
X : parts per Million : Proton
D)
δNHb=10.690pm
δNHa=10.400ppm
10.680
10.400
X : parts per Million : Proton
abundance

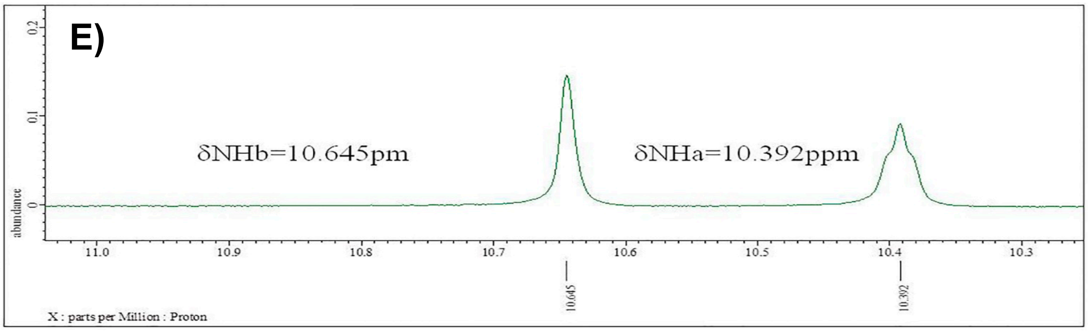

Supplementary Figure S6. The chemical shifts of amide protons in g-NOP **7** at 293K (A), 303K (B), 313K (C), 323K (D), and 333K (E).

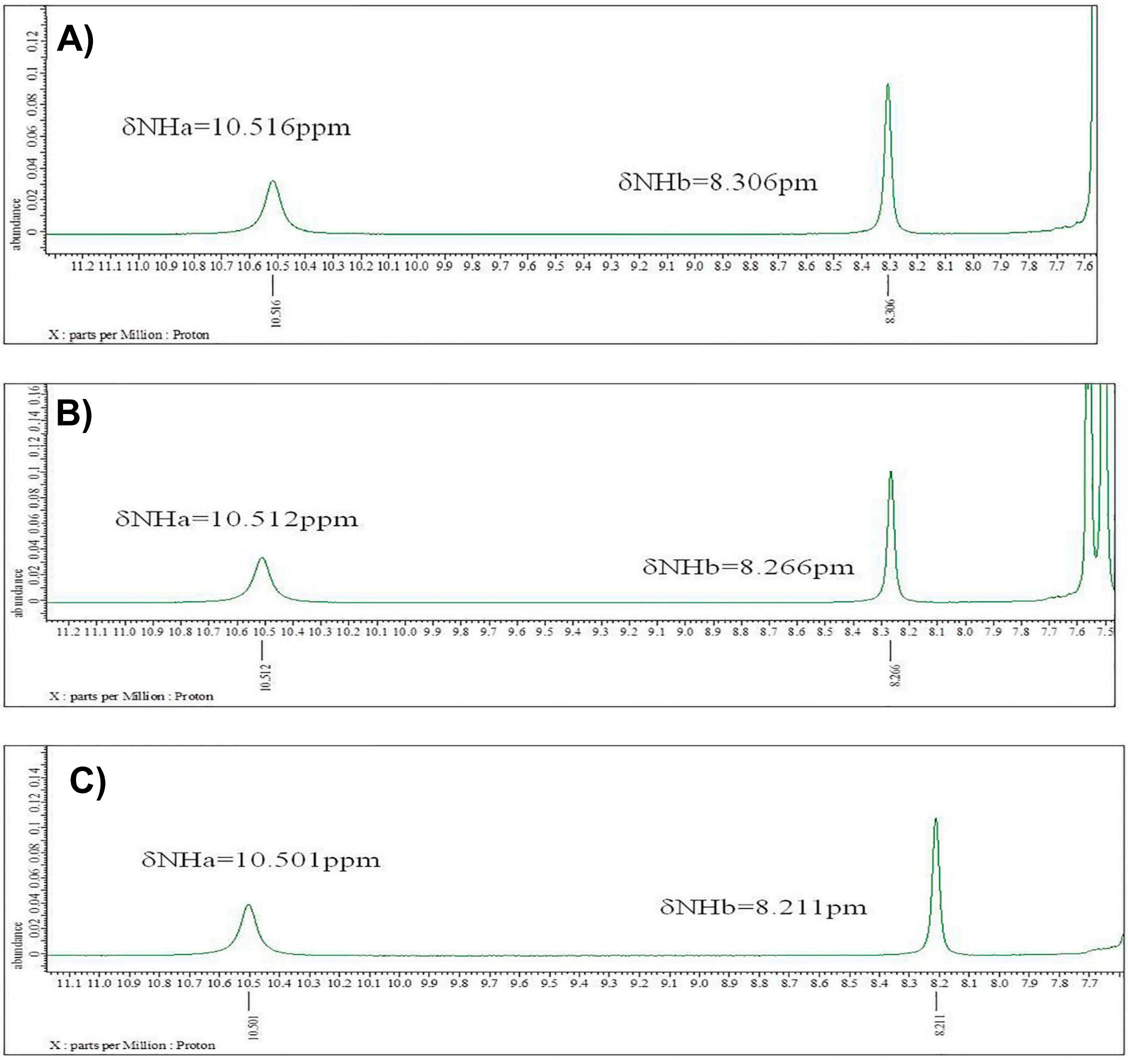

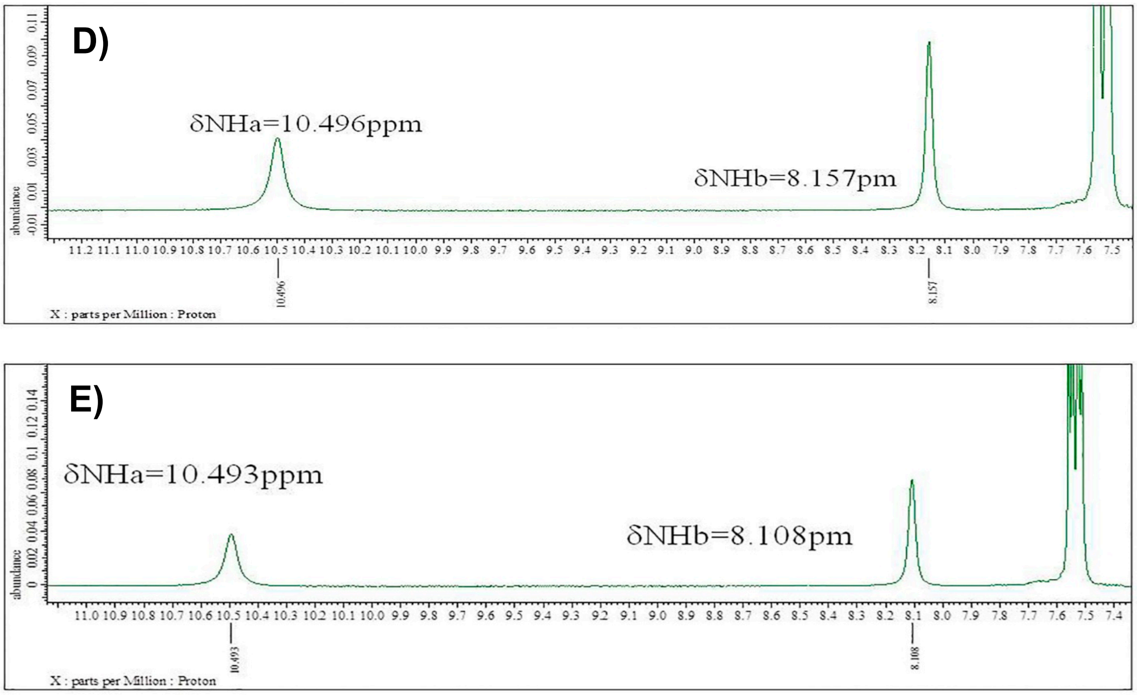

Supplementary Figure S7. The chemical shifts of amide protons in g-NOP **9** at 293K (A), 303K (B), 313K (C), 323K (D), and 333K (E).

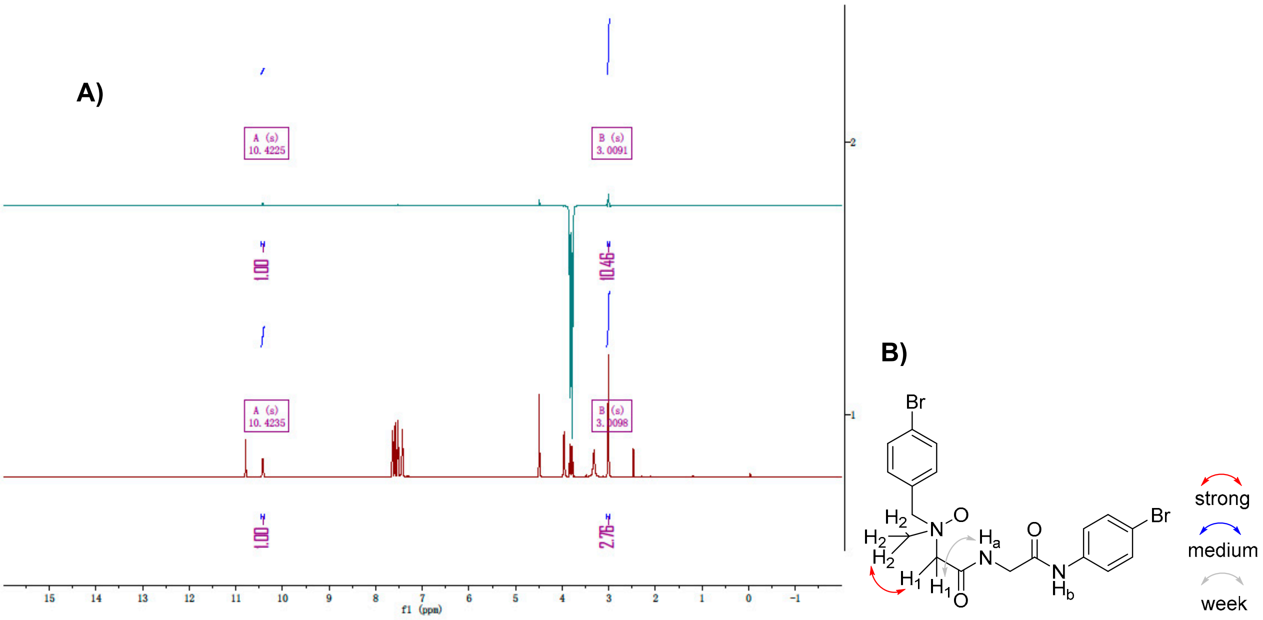

Supplementary Figure S8. A) The arrows represent observed intermolecular NOEs; B) The structures of g-NOP **7**.

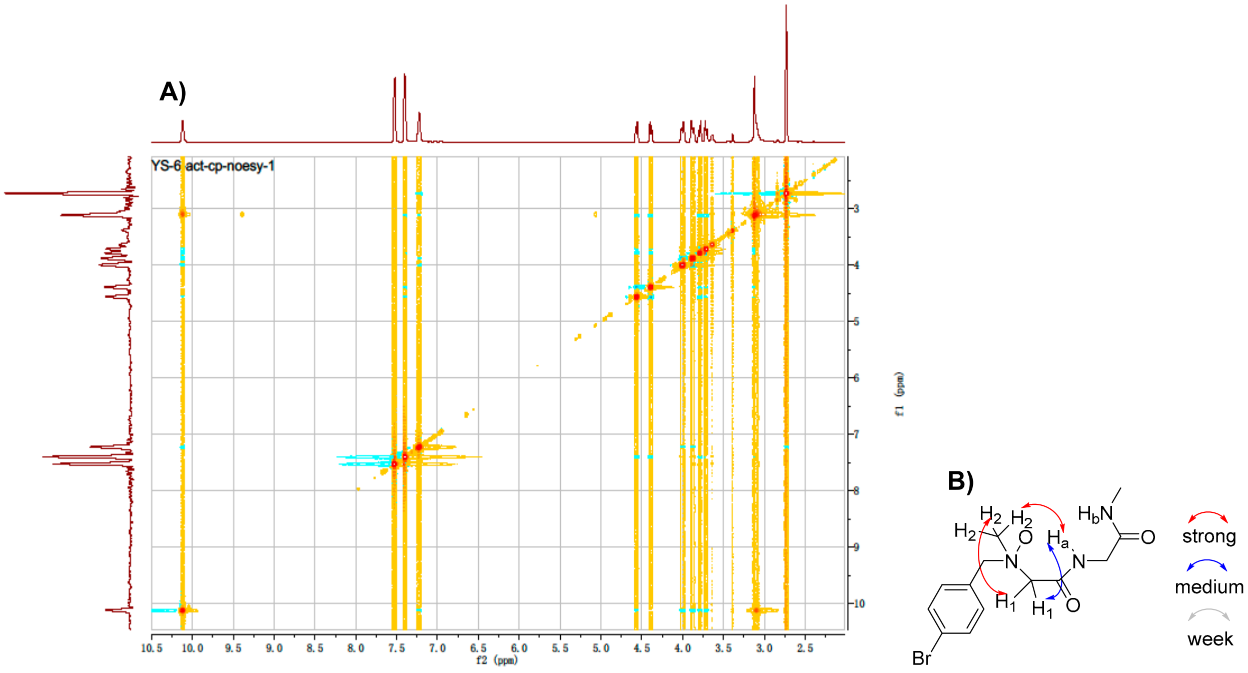

Supplementary Figure S9. A) The arrows represent observed intermolecular NOEs. B) The structures of g-NOP **9**.

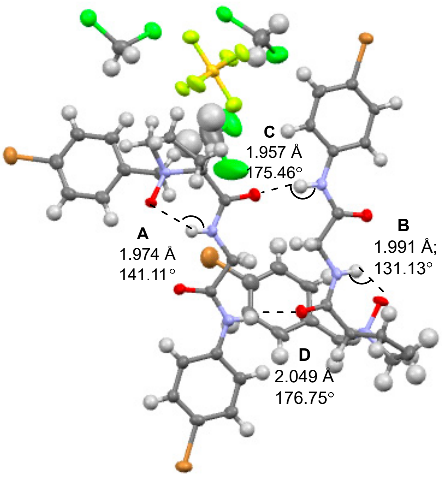

Supplementary Figure S10. X-ray structure of p-NOP **5**.

Table S1. Measurements for p-NOP **5** H-bonds

H-Bond	N-H··O-N distance	N-H···O angle
A	1.974Å	141.11°
B	1.991Å	131.13°

C	1.957Å	175.46°
D	2.049Å	176.75°

Table S2. Crystal data and structure refinement for p-NOP **5**

Identification code	5
Empirical formula	$C_{20}H_{21}Br_2N_3O_3$
Formula weight	1423.27
Temperature/K	100
Crystal system	monoclinic
Space group	$P2_1$
a/Å	12.4592(2)
b/Å	17.5683(2)
c/Å	13.26226(17)
α/°	90
β/°	105.5619(15)
γ/°	90
Volume/Å^3	2796.52(7)
Z	2
ρ_{calc}g/cm^3	1.690
μ/mm^{-1}	7.106
F(000)	1416.0
Crystal size/mm^3	0.22 × 0.19 × 0.16
Radiation	CuKα (λ = 1.54184)
2Θ range for data collection/°	6.918 to 146.966
Index ranges	-15 ≤ h ≤ 13, -21 ≤ k ≤ 21, -16 ≤ l ≤ 16
Reflections collected	62029
Independent reflections	11093 [R_{int} = 0.0766, R_{sigma} = 0.0385]
Data/restraints/parameters	11093/7/653
Goodness-of-fit on F^2	1.061
Final R indexes [I>=2σ (I)]	R_1 = 0.0817, wR_2 = 0.2155
Final R indexes [all data]	R_1 = 0.0829, wR_2 = 0.2167
Largest diff. peak/hole / e Å^{-3}	1.32/-1.08
Flack parameter	0.02(3)

Table S3. Calculated Energies and NHa of p-NOP **5** and g-NOP **7**

	Gas phase				Solvent($CHCl_3$)		NHa (ppm)	
	B3LYP/6-31G(d,p) (hartree)	ΔE (Kcal/mol)	MP2/6-31G(d,p) (hartree)	ΔE (Kcal/mol)	B3LYP/6-31G(d,p)	ΔE (Kcal/mol)	Cal.	Exp.
**5a**	-6307.785992	8.39	-6306.399085	13.46	-6306.747987	3.15	10.48	10.65
**5b**	-6307.799360	0	-6306.420538	0	-6306.753001	0	10.21	
**7a**	-6230.089109	0	-6229.467044	0	-6230.775777	0	11.11	10.79
**7b**	-6230.065791	14.63	-6229.446507	12.89	-6230.775413	0.22	11.02	
**7c**	-6230.054363	21.8	-6229.444006	14.46	-6230.775321	9.28	11.78	

Table S4. Geometries (Cartesian coordinates) of p-NOP **5** and g-NOP **7** optimized using B3LYP/6-31G (d, p)

**5a**				**5b**			
Br	-3.332	6.274	8.9887	Br	6.3192	15.1202	1.8187
Br	5.1359	10.74	19.7688	Br	4.947	9.0345	10.7897
O	0.4311	11.7655	10.3614	O	8.7212	14.088	8.3492
O	4.0452	13.308	8.9803	O	7.1966	13.4977	12.8617
O	2.6476	11.6197	13.2424	O	10.8494	12.8723	11.082
N	0.7404	11.7427	8.9867	N	6.5313	13.8983	7.7525
N	2.962	12.5543	10.7894	H	5.7397	13.7156	8.0898
N	4.7736	12.224	13.9029	N	8.2926	13.5592	10.8903
H	5.5169	12.5754	13.5874	H	9.086	13.5996	10.5109
C	-1.9025	7.5069	8.9164	N	10.6666	12.5736	12.4541
C	-1.5307	8.0182	7.6886	C	5.2267	14.3779	4.3439
H	-1.9906	7.7511	6.9016	H	4.3947	14.3392	3.8865
C	-0.4808	8.9212	7.6094	C	6.3908	14.7012	3.6757
H	-0.2141	9.262	6.7637	C	7.6142	14.7029	4.3068
C	0.1789	9.3305	8.7529	H	8.4025	14.8839	3.8073
C	-0.1878	8.7666	9.9935	C	7.7064	14.4411	5.6624
H	0.2833	9.0143	10.7805	H	8.5484	14.4622	6.1019
C	-1.2194	7.8601	10.0739	C	6.5525	14.1513	6.3702
H	-1.4624	7.4806	10.9108	C	5.3075	14.1109	5.7135
C	1.2592	10.353	8.6673	H	4.5209	13.9018	6.2028
H	1.6416	10.3495	7.7551	C	7.5525	13.8948	8.6354
H	1.982	10.1193	9.3023	C	7.1233	13.5908	10.0471
C	-0.4456	12.145	8.1588	H	6.4961	14.2865	10.3665
H	-1.2839	11.8112	8.5651	H	6.6608	12.7159	10.0791
H	-0.3708	11.7813	7.2415	C	8.2242	13.4714	12.2229
C	-0.4259	13.6734	8.1371	C	9.56	13.4749	12.9652
H	-0.5193	14.009	7.2108	H	9.3697	13.1973	13.908
H	-1.1692	14.0388	8.6813	C	10.2258	14.854	13.0303
C	0.9218	14.088	8.7248	H	10.0506	15.3635	12.2012
H	1.3337	14.8118	8.1895	H	9.8792	15.3705	13.8007
H	0.8196	14.3955	9.66	C	11.7222	14.587	13.1913
C	1.7601	12.8161	8.6584	H	12.2278	14.9805	12.4362
H	2.0355	12.6878	7.7053	H	12.0564	14.9822	14.0358

C	3.0307	12.8776	9.5003	C	11.8851	13.0673	13.2105
C	4.1028	12.6632	11.6543	H	12.7216	12.7915	12.7569
H	4.3752	13.6119	11.7323	H	11.8921	12.7212	14.138
H	4.8623	12.1537	11.2762	C	10.4465	11.0944	12.6675
C	3.7474	12.1186	13.0201	H	11.1675	10.599	12.2037
C	4.8011	11.8428	15.2585	H	10.526	10.9011	13.6346
C	6.0211	11.9552	15.9062	C	9.1396	10.5849	12.1884
H	6.7844	12.2504	15.4246	C	8.8972	10.3126	10.8265
C	6.1354	11.6443	17.2452	H	9.5825	10.4654	10.1864
H	6.9602	11.7549	17.7013	C	7.6656	9.8224	10.4125
C	5.0164	11.1647	17.9121	H	7.5197	9.5976	9.5003
C	3.787	11.0311	17.2937	C	6.6596	9.6643	11.3362
H	3.0301	10.7237	17.7792	C	6.8671	9.9085	12.6713
C	3.6805	11.3579	15.9445	H	6.17	9.7557	13.2999
H	2.8521	11.2543	15.4923	C	8.0996	10.3794	13.0955
H	2.1801	12.3681	11.0513	H	8.238	10.5656	14.0166

7a				7b			
N	-4.48057	-2.54542	1.99164	N	-10.9677	-12.4923	1.965502
C	-5.01915	-3.55235	2.890041	C	-11.2026	-12.081	0.6071
C	-6.54939	-3.61317	3.005483	C	-12.6983	-11.8399	0.313489
N	-7.23378	-2.81984	2.126542	N	-12.9165	-11.0645	-0.79705
O	-7.0602	-4.34731	3.843683	O	-13.5836	-12.3457	0.991148
C	-8.6258	-2.66604	1.968711	C	-14.1359	-10.7292	-1.42494
C	-9.56801	-3.35744	2.745832	C	-15.3792	-11.2267	-1.00617
C	-10.931	-3.14912	2.529329	C	-16.5388	-10.8605	-1.69118
C	-11.3555	-2.25841	1.545974	C	-16.464	-10.0016	-2.78523
C	-10.4291	-1.56569	0.766994	C	-15.2347	-9.49802	-3.20931
C	-9.0691	-1.77081	0.981971	C	-14.0766	-9.86317	-2.52828
Br	-13.2202	-1.98255	1.26084	Br	-18.0569	-9.5037	-3.70634
C	1.326468	1.251065	3.87986	C	-13.4001	-17.5659	7.266598
C	1.802614	0.585211	2.752016	C	-13.7905	-16.3485	7.820488
C	0.935269	-0.25215	2.04783	C	-13.1493	-15.1815	7.399843
C	-0.39119	-0.43358	2.461612	C	-12.1263	-15.2219	6.44344
C	-0.8421	0.238553	3.60824	C	-11.7449	-16.4626	5.909264
C	0.010734	1.085418	4.315317	C	-12.3825	-17.6349	6.313956
Br	2.49643	2.401102	4.84908	Br	-14.2642	-19.1686	7.832428
C	-1.29109	-1.368	1.699895	C	-11.4321	-13.952	6.031609
C	-3.17154	-1.69693	0.110434	C	-10.997	-12.1897	4.388693
C	-3.80327	-2.87995	0.860791	C	-11.0944	-11.6035	2.975173

O	-3.7167	-4.0092	0.38617	O	-11.2298	-10.3883	2.829569
N	-2.46198	-0.67271	0.983115	N	-11.6805	-13.5343	4.577771
C	-1.95724	0.427106	0.09446	C	-13.1572	-13.4067	4.305898
O	-3.33988	-0.12822	1.89943	O	-11.1232	-14.478	3.736532
H	-4.29488	-1.57042	2.294952	H	-10.9814	-13.4822	2.269806
H	-4.68642	-4.5378	2.5516	H	-10.6271	-11.1737	0.391939
H	-4.63994	-3.39843	3.905327	H	-10.8513	-12.8741	-0.06199
H	-6.64758	-2.27122	1.509474	H	-12.0938	-10.6501	-1.21421
H	-9.23086	-4.04711	3.507002	H	-15.4325	-11.8878	-0.15288
H	-11.6564	-3.68497	3.1315	H	-17.4976	-11.2472	-1.36426
H	-10.7616	-0.87286	0.002162	H	-15.1779	-8.82755	-4.05941
H	-8.34686	-1.22882	0.376447	H	-13.1189	-9.46888	-2.85994
H	2.828768	0.715795	2.427791	H	-14.5785	-16.3088	8.563941
H	1.305693	-0.77433	1.169021	H	-13.4523	-14.23	7.830005
H	-1.87183	0.115157	3.922891	H	-10.9663	-16.4928	5.155812
H	-0.34317	1.608103	5.196824	H	-12.0902	-18.5912	5.894647
H	-0.72366	-1.91642	0.942354	H	-11.7437	-13.1209	6.671894
H	-1.77559	-2.08474	2.363886	H	-10.3471	-14.0609	6.083323
H	-2.46983	-2.10748	-0.61771	H	-9.94659	-12.3557	4.63995
H	-3.95492	-1.13777	-0.40674	H	-11.4327	-11.4584	5.072045
H	-1.49328	1.174906	0.733235	H	-13.5926	-14.3933	4.446015
H	-2.825	0.858046	-0.40234	H	-13.2864	-13.0891	3.271568
H	-1.2409	0.034184	-0.63288	H	-13.6026	-12.6825	4.994181

7c			
N	-4.0652	-1.03722	4.836192
C	-5.1888	-1.06835	3.939083
C	-6.24333	-2.11851	4.34712
N	-7.15142	-2.39014	3.355905
O	-6.27297	-2.6112	5.467906
C	-8.30466	-3.20472	3.400172
C	-8.73878	-3.862	4.561225
C	-9.8985	-4.63796	4.525575
C	-10.6219	-4.76189	3.34156
C	-10.1991	-4.11564	2.180451
C	-9.04348	-3.33997	2.214181
Br	-12.2008	-5.82946	3.305337
C	-5.07683	-2.79472	11.66877
C	-4.20406	-3.80469	11.26932
C	-3.72573	-3.7995	9.957334
C	-4.11377	-2.80718	9.047485
C	-5.00505	-1.81006	9.472664

C	-5.48353	-1.79654	10.78253
Br	-5.73542	-2.78372	13.45926
C	-3.61132	-2.8421	7.630449
C	-2.06993	-1.94558	5.896898
C	-3.10914	-1.99224	4.770329
O	-3.0007	-2.82288	3.868421
N	-2.63873	-1.6968	7.285806
C	-1.49374	-1.66351	8.254599
O	-3.28205	-0.47543	7.330192
H	-4.07889	-0.52484	5.737069
H	-5.66995	-0.08374	3.942958
H	-4.83544	-1.27124	2.922255
H	-6.96811	-1.96007	2.459033
H	-8.17059	-3.76772	5.475683
H	-10.2305	-5.14439	5.425113
H	-10.7617	-4.21486	1.259058
H	-8.71505	-2.83688	1.30774
H	-3.90282	-4.5799	11.96491
H	-3.04676	-4.58787	9.641322
H	-5.3009	-1.0362	8.774421
H	-6.16789	-1.02145	11.10883
H	-3.08537	-3.78105	7.432673
H	-4.43178	-2.73298	6.918884
H	-1.52861	-2.89319	5.886267
H	-1.37914	-1.11828	5.718032
H	-1.90363	-1.43273	9.235086
H	-0.83043	-0.86001	7.937615
H	-0.97148	-2.62481	8.265246

Table S5. Laplacians of the electron density, ∇2 ρ (r), charge densities, ρ (r), and Lagrangian kinetic energies, G(r), at each C=O···Ha BCP, and calculated E_{HB} energies for **5** and **7.**

**Parameters**	**5**	**7**
$\nabla^2 \rho$ (r) (a.u.)	0.12805	0.13075

Parameters	5	7
ρ(r) (a.u.)	0.03934	0.04232
G(r) (a.u.)	0.03326	0.03521
E_{HB} (kcal mol^{-1})[a]	8.9	9.5

[a]$E_{HB} = 0.429 \times G(r) \times 627.51$

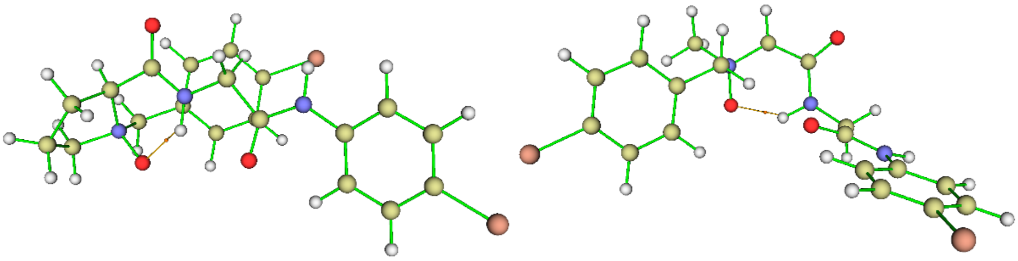

Figure S11 Interatomic bond critical points (orange circles) identified by QTAIM and the corresponding paths between the concerned atoms (left: **5**; right: **7**). Circles in yellow correspond to ring critical points.

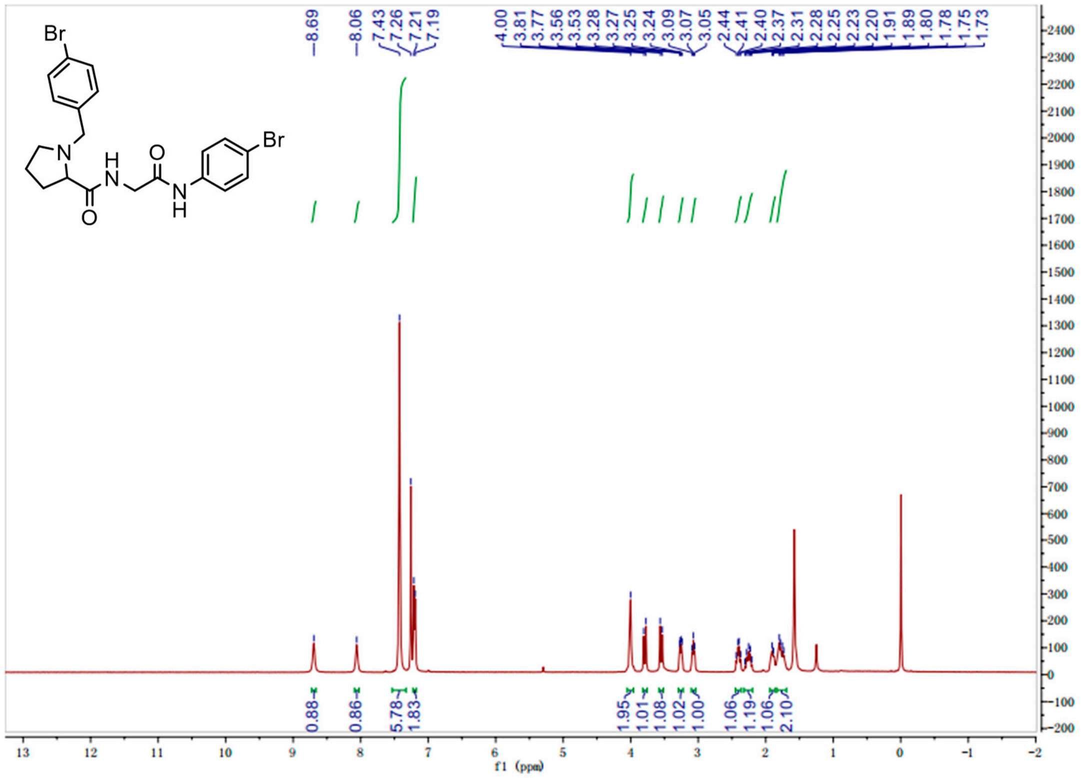

Supplementary Figure S12. ^{1}H NMR spectra of pepetide **4** ($CDCl_3$).

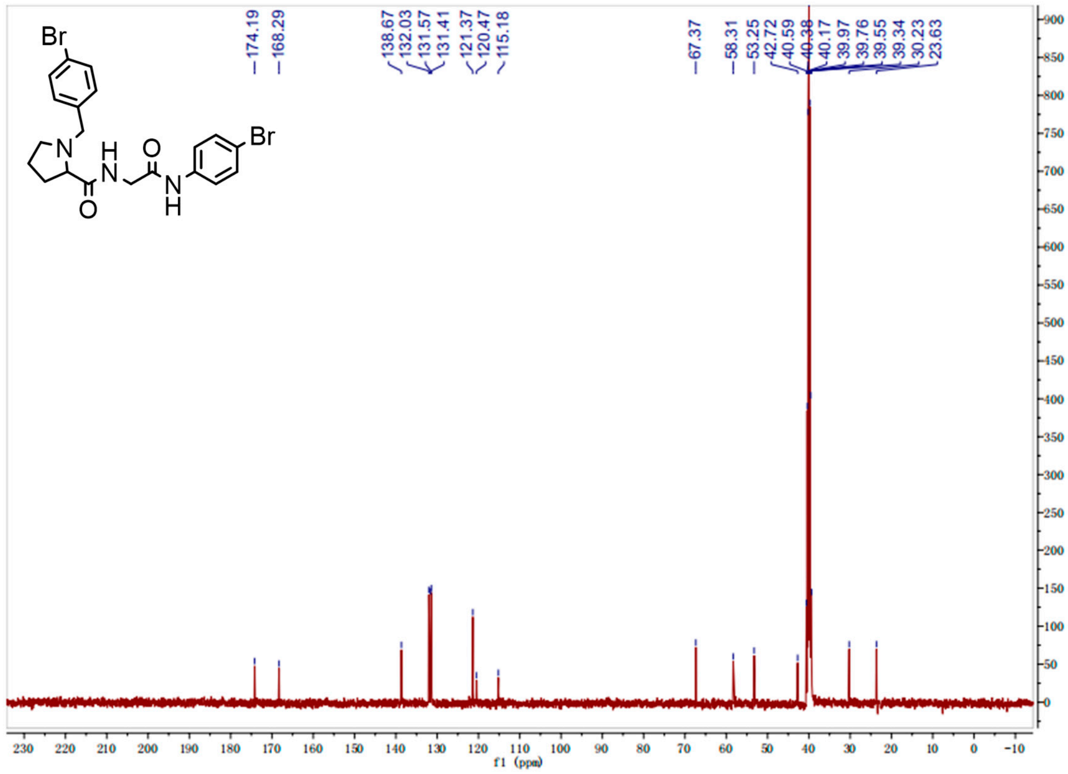

Supplementary Figure S13. ^{13}C NMR spectra of pepetide **4** (DMSO-d_6).

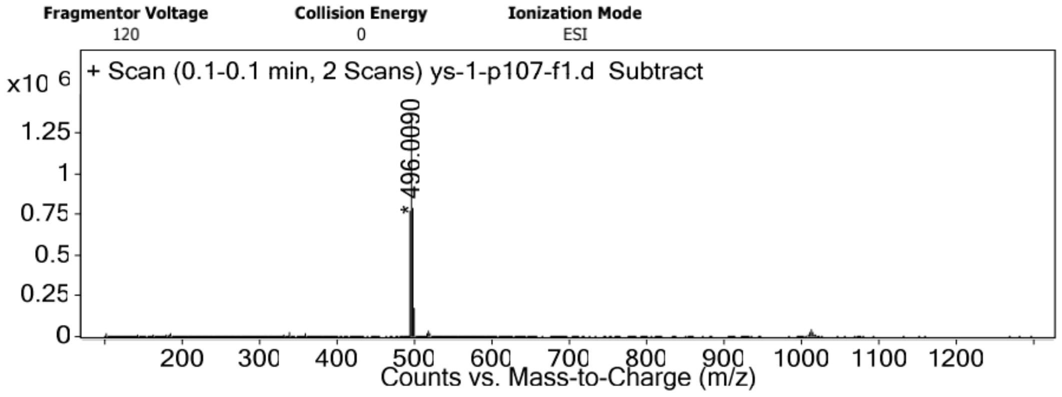

Supplementary Figure S14. HRMS (ESI-TOF) spectra of peptide **4**.

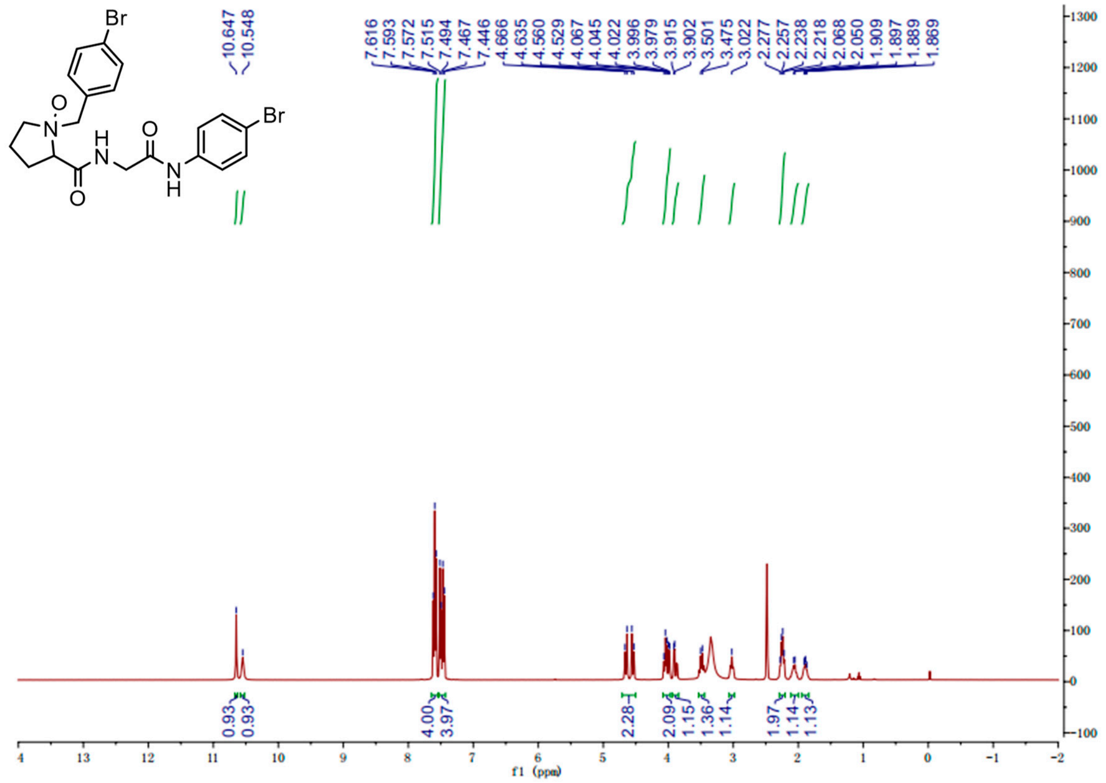

Supplementary Figure S15. ^{1}H NMR spectra of p-NOP **5** ($CDCl_3$).

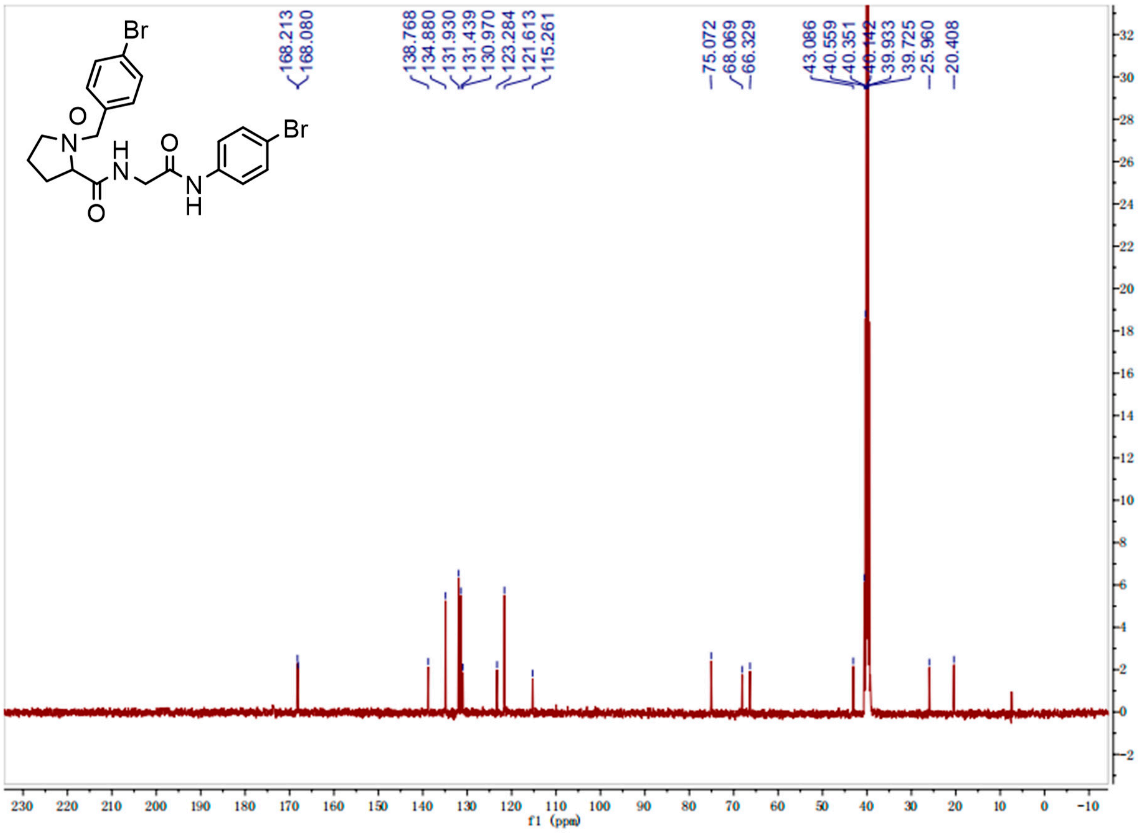

Supplementary Figure S16. ^{13}C NMR spectra of p-NOP **5** ($CDCl_3$).

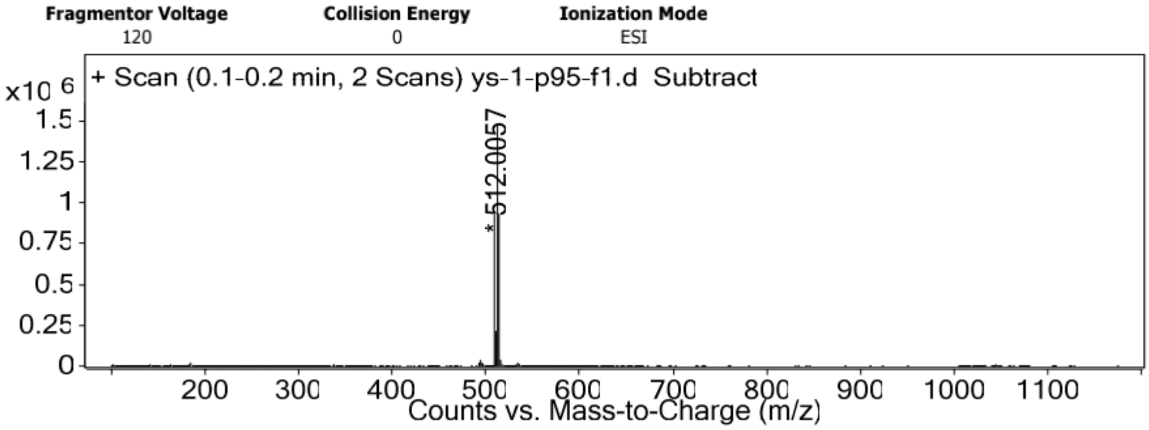

Supplementary Figure S17. HRMS (ESI-TOF) spectra of p-NOP **5**.

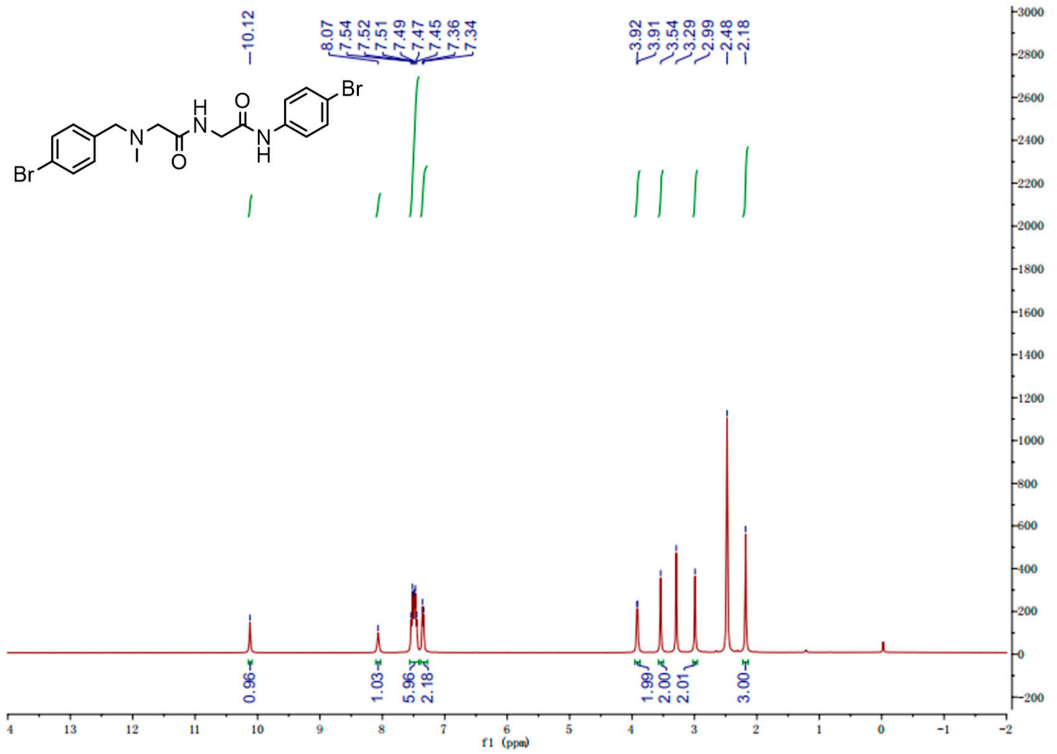

Supplementary Figure S18. ^{1}H NMR spectra of peptide **6** ($CDCl_3$).

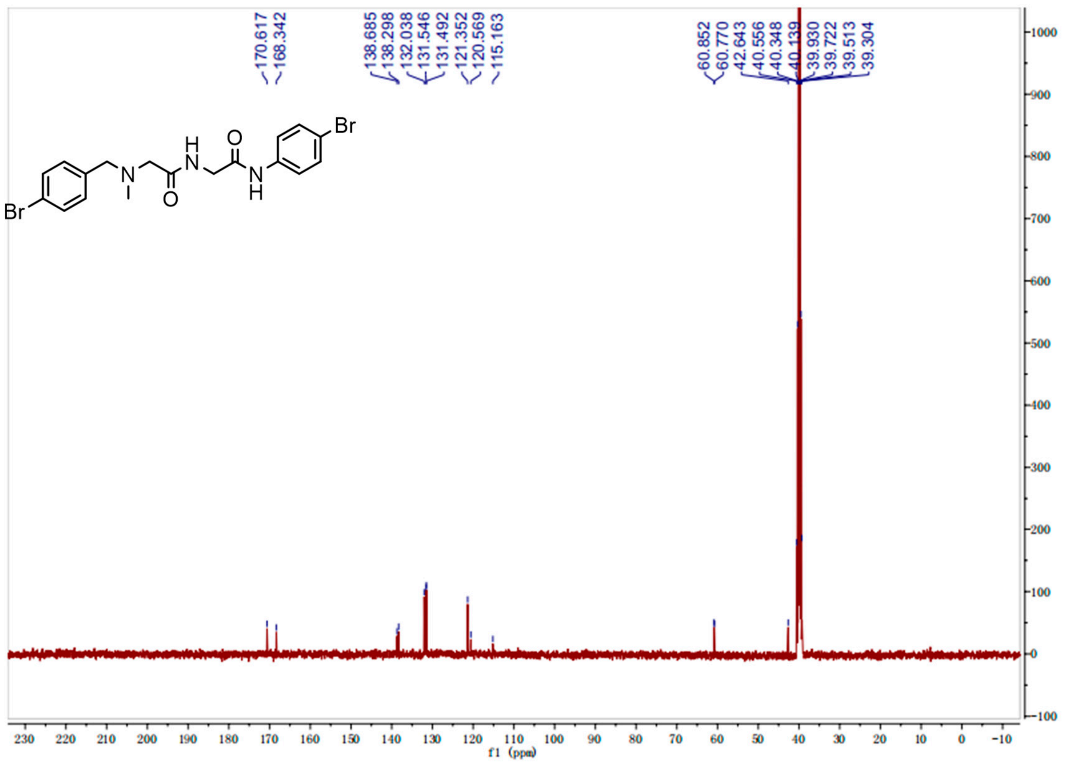

Supplementary Figure S19. ^{13}C NMR spectra of peptide **6** (DMSO-d_6).

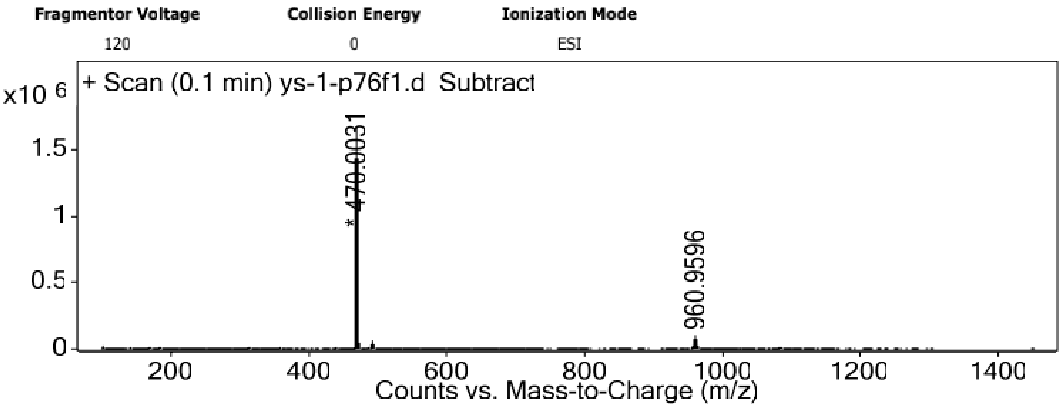

Supplementary Figure S20. HRMS (ESI-TOF) spectra of peptide **6**.

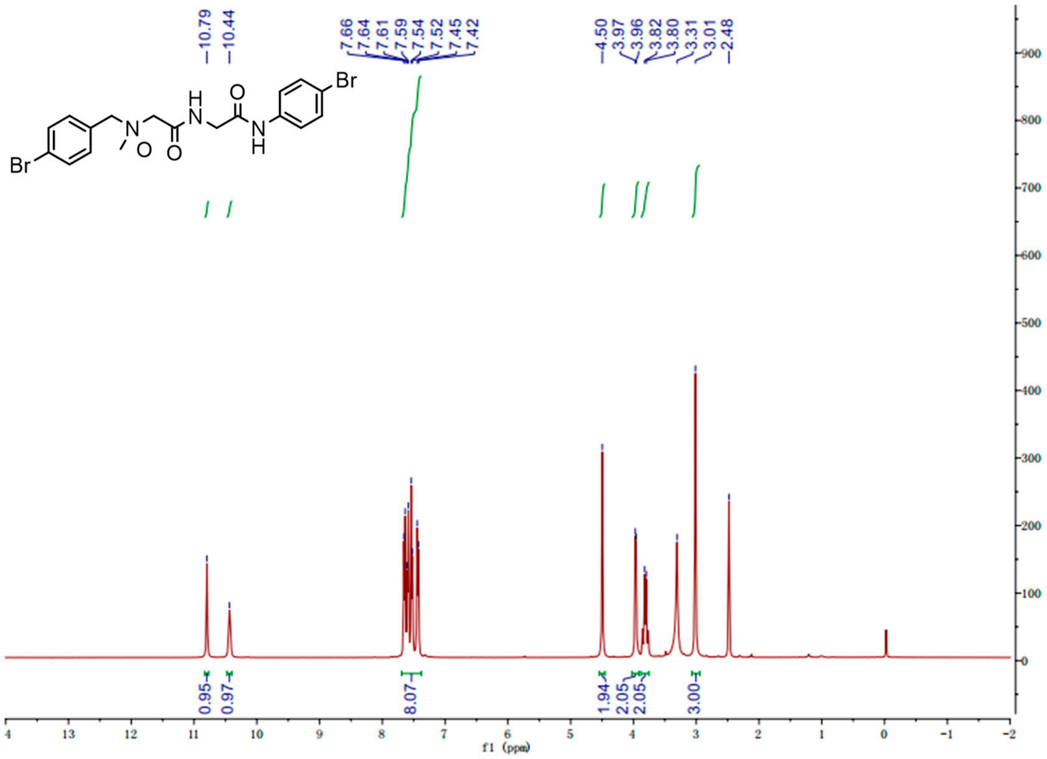

Supplementary Figure S21. ^{1}H NMR spectra of g-NOP **7** (DMSO-d_6).

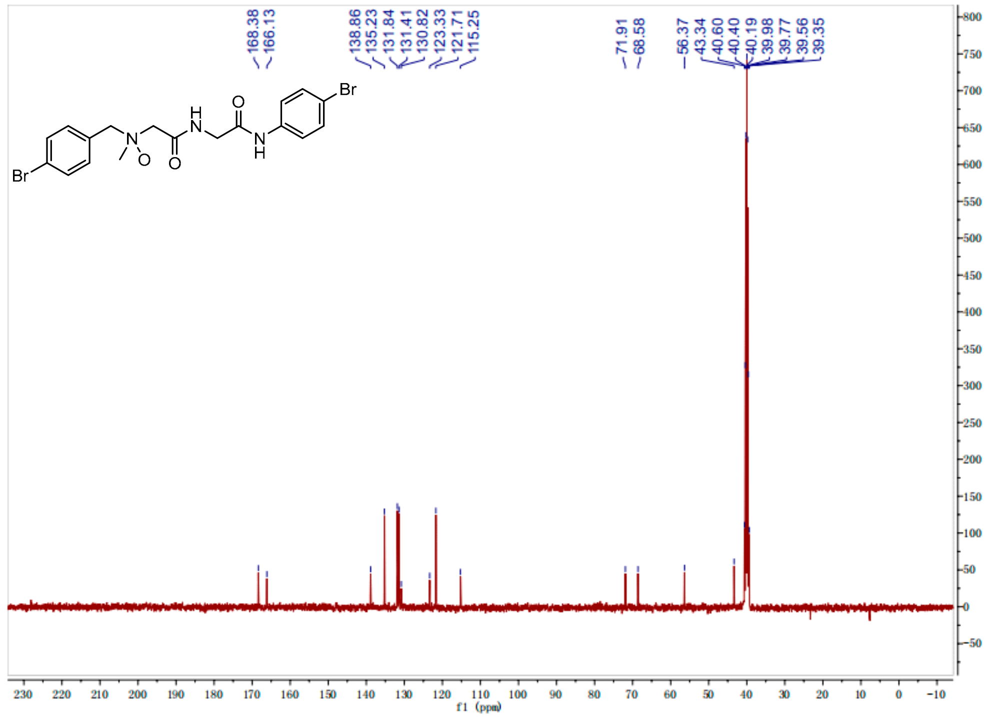

Supplementary Figure S22. ^{13}C NMR spectra of g-NOP **7** (DMSO-d_6).

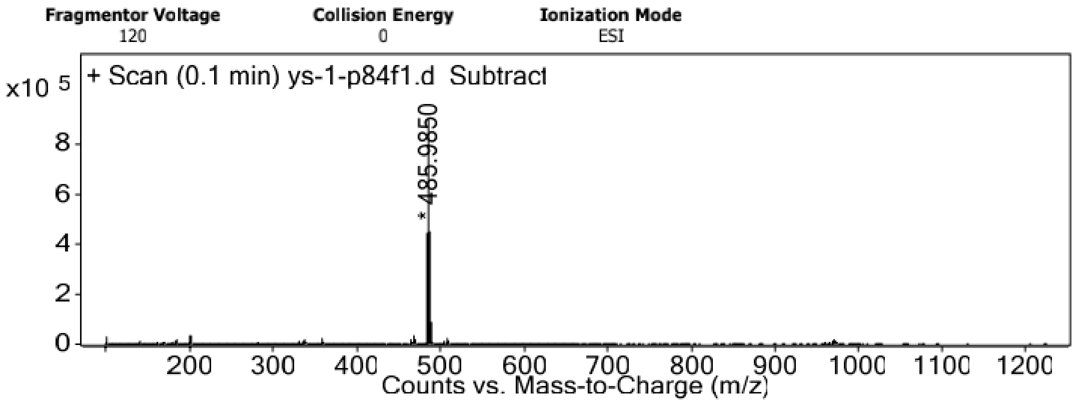

Supplementary Figure S23. HRMS (ESI-TOF) spectra of g-NOP **7**.

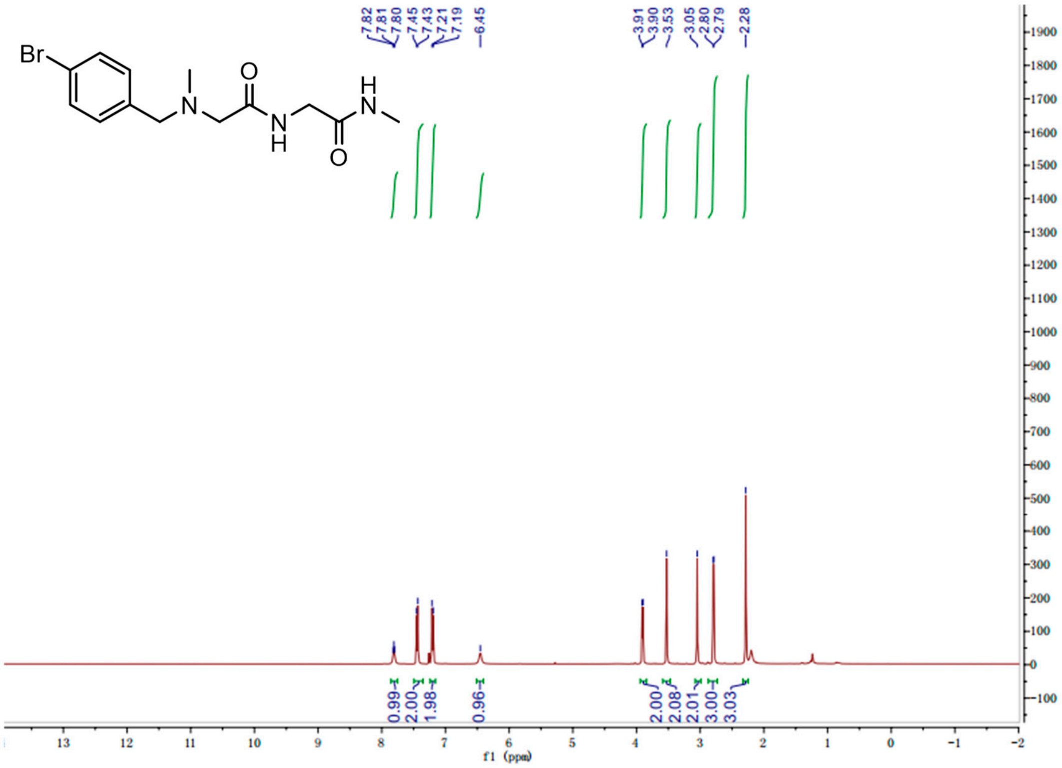

Supplementary Figure S24. ^{1}H NMR spectra of peptide **8** ($CDCl_3$).

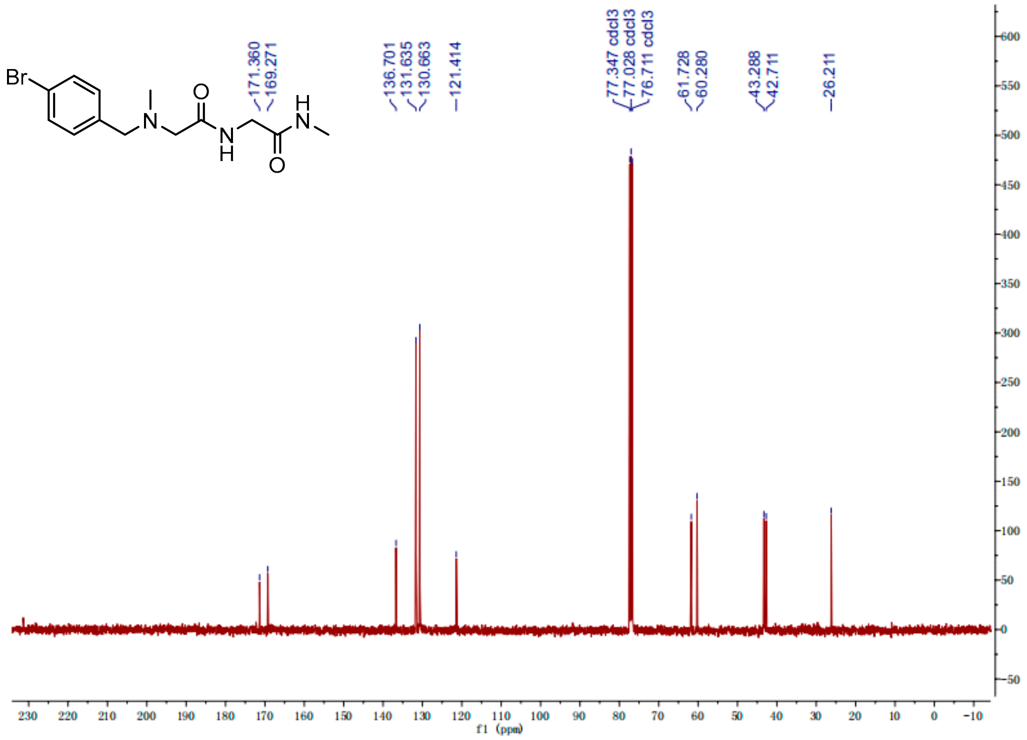

Supplementary Figure S25. ^{13}C NMR spectra of peptide **8** ($CDCl_3$).

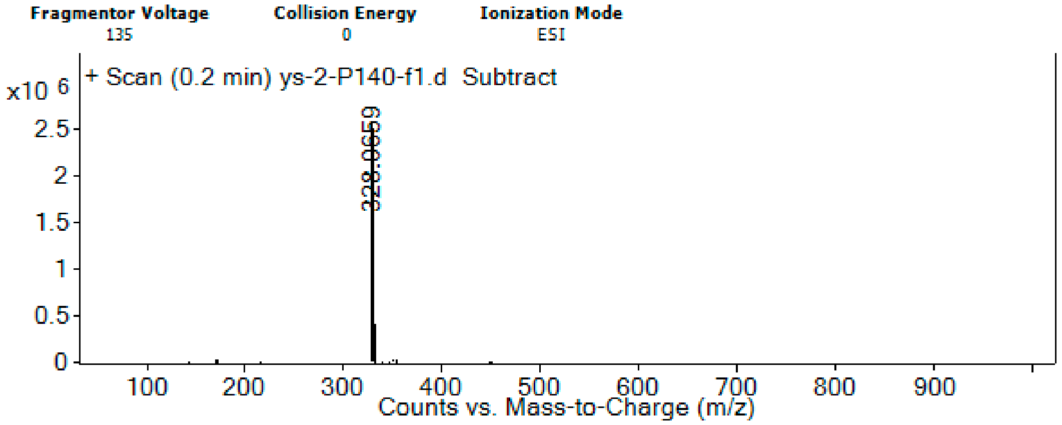

Supplementary Figure S26. HRMS (ESI-TOF) spectra of peptide **8**.

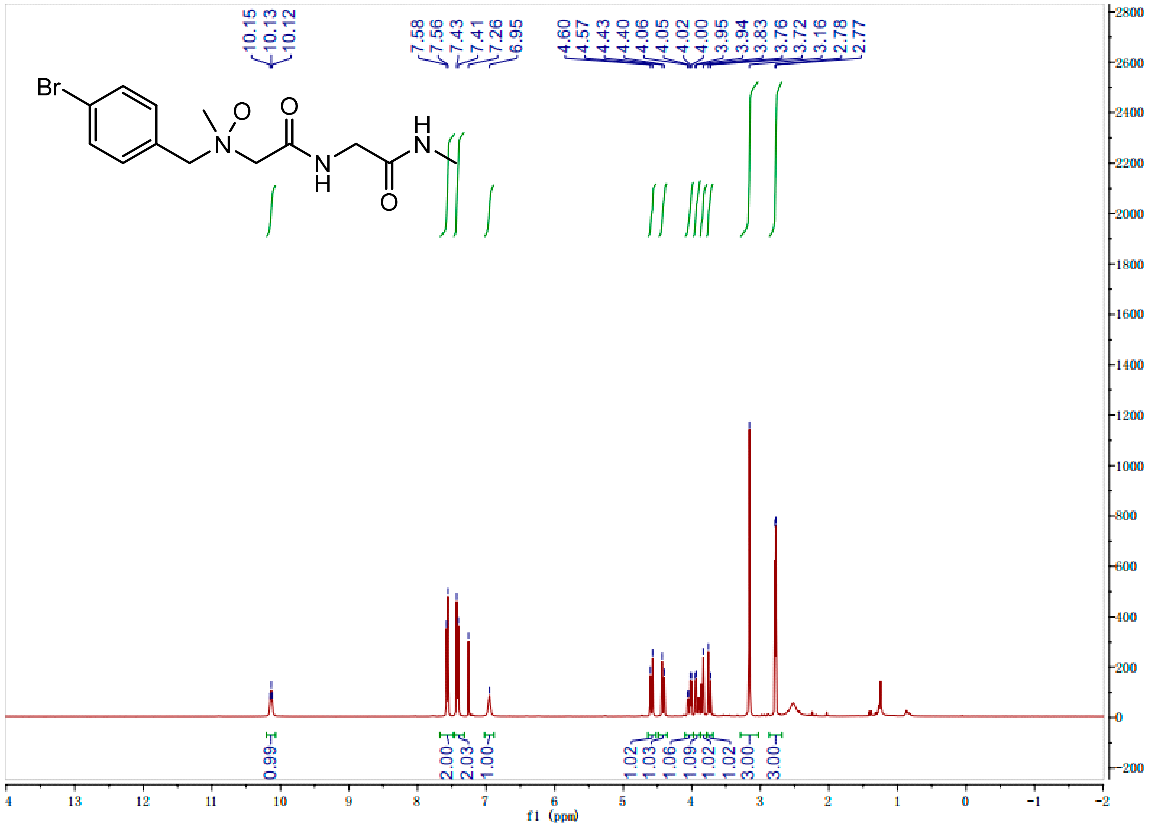

Supplementary Figure S27. ^{1}H NMR spectra of g-NOP **9** ($CDCl_3$).

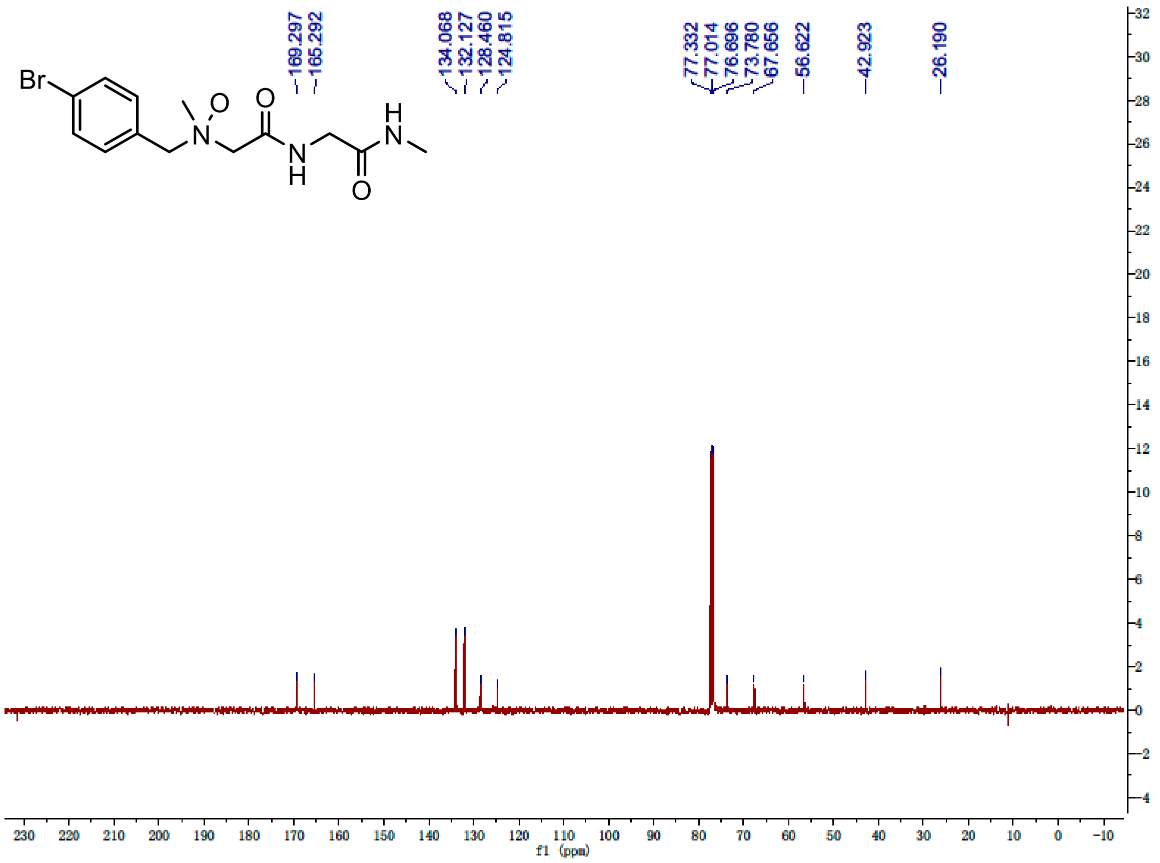

Supplementary Figure S28. ^{13}C NMR spectra of g-NOP **9** ($CDCl_3$).

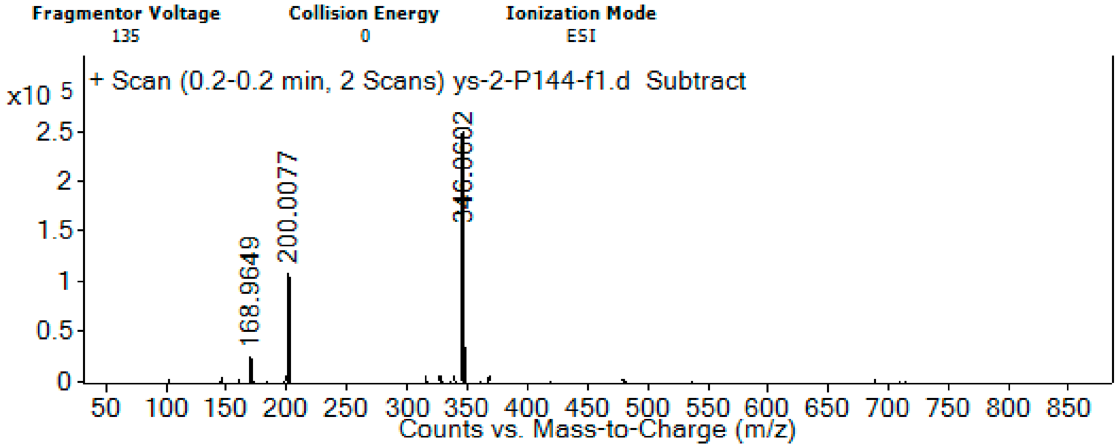

Supplementary Figure S29. HRMS (ESI-TOF) spectra of g-NOP **9**.

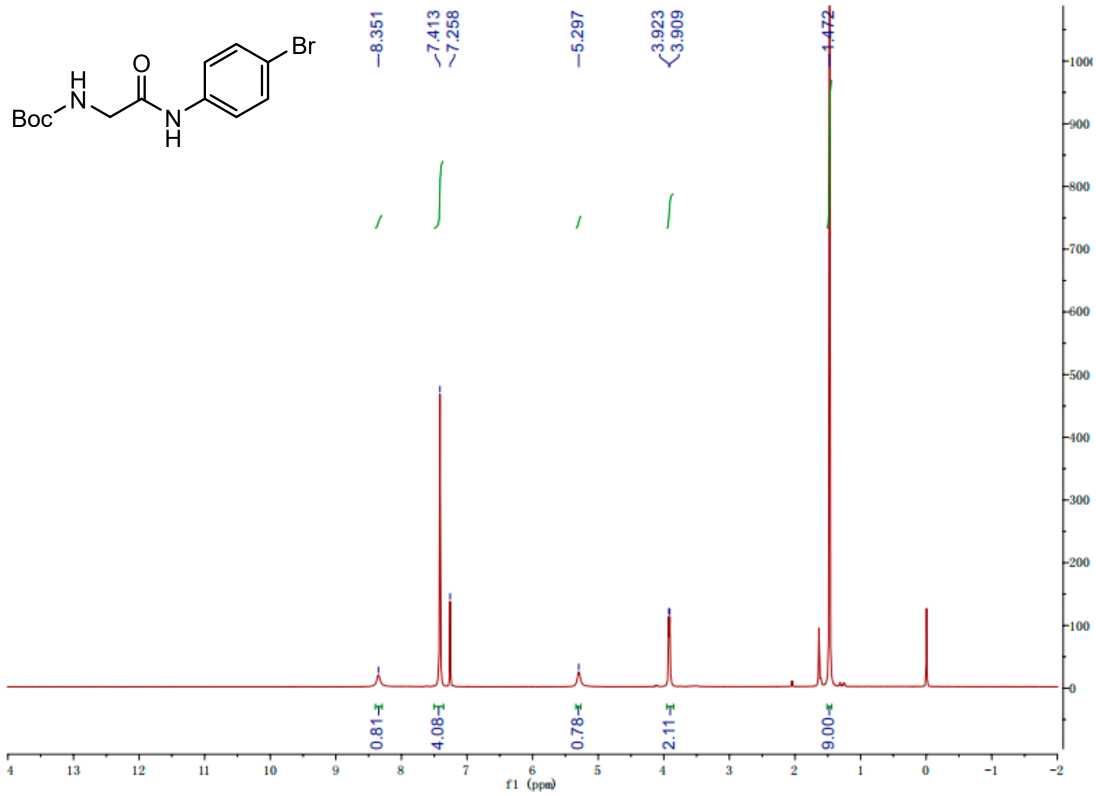

Supplementary Figure S30. ^{1}H NMR spectra of compound **1s** ($CDCl_3$).

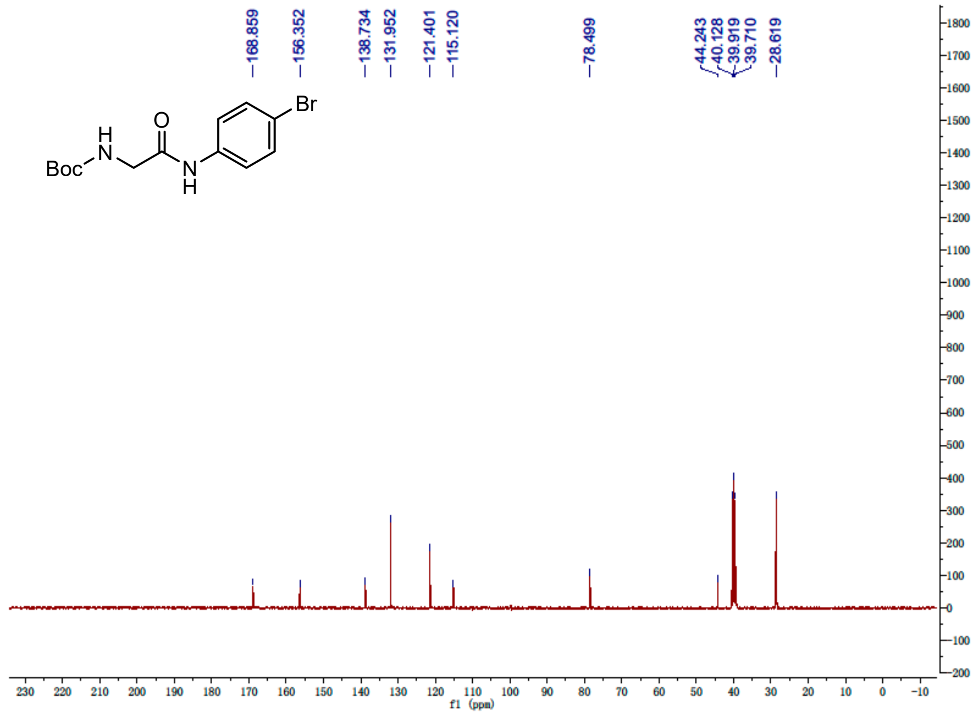

Supplementary Figure S31. ^{13}C NMR spectra of compound **1s** (DMSO-d_6).

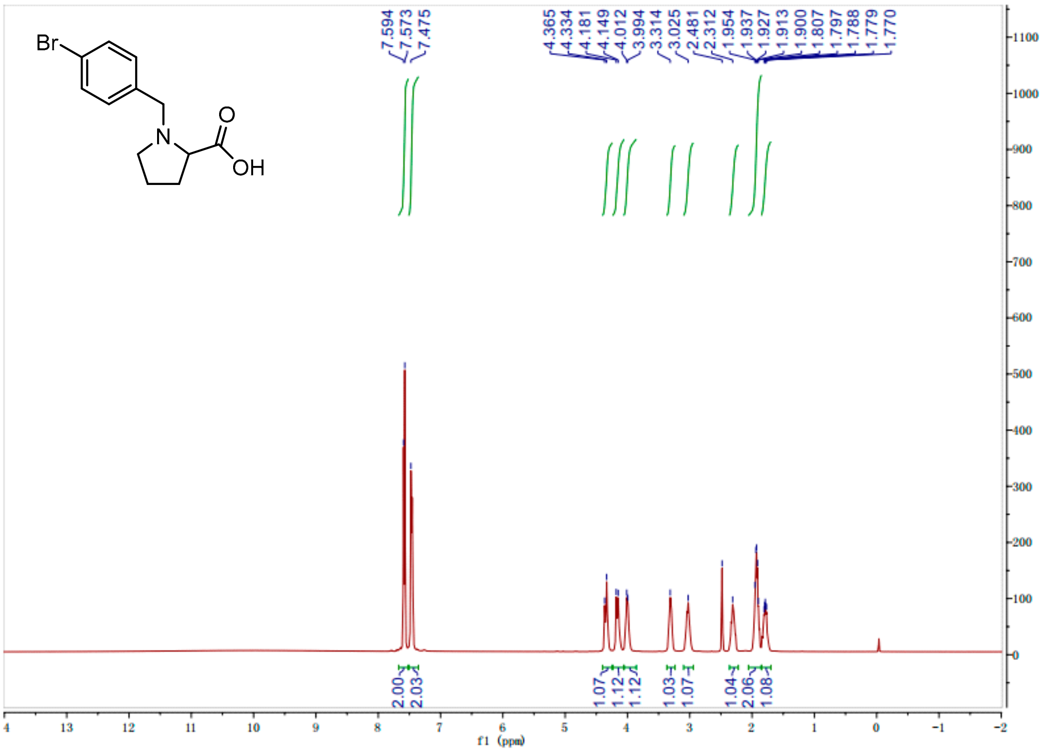

Supplementary Figure S32. ^{1}H NMR spectra of compound **2s** (DMSO-d_6).

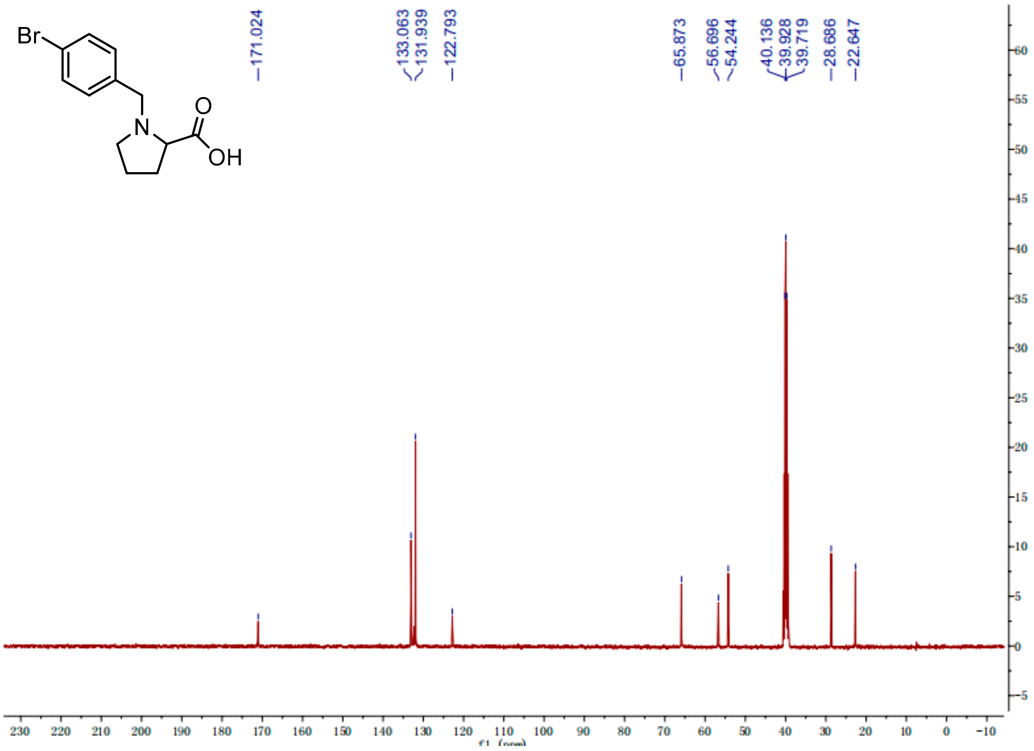

Supplementary Figure S33. ^{13}C NMR spectra of compound **2s** (DMSO-d_6).

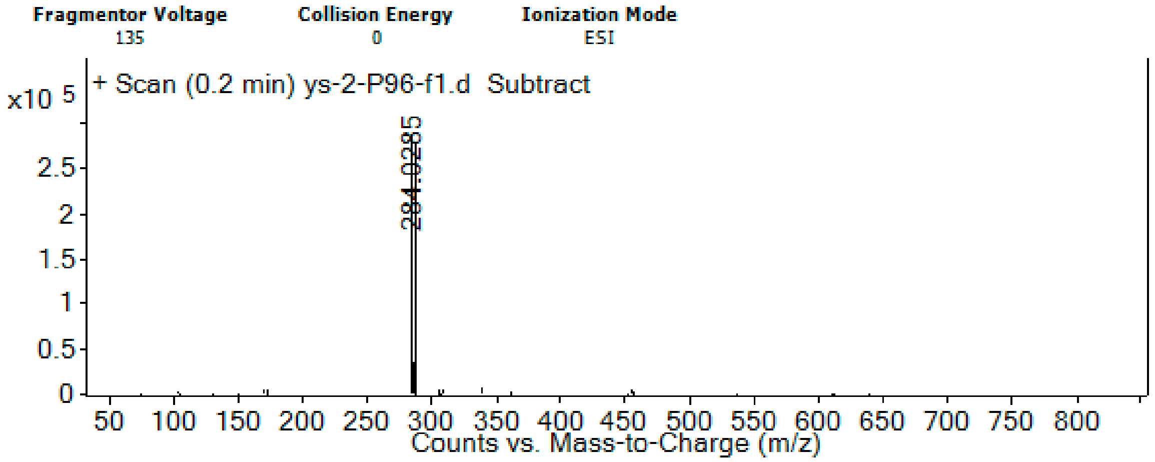

Supplementary Figure S34. HRMS (ESI-TOF) spectra of compound **2s**.

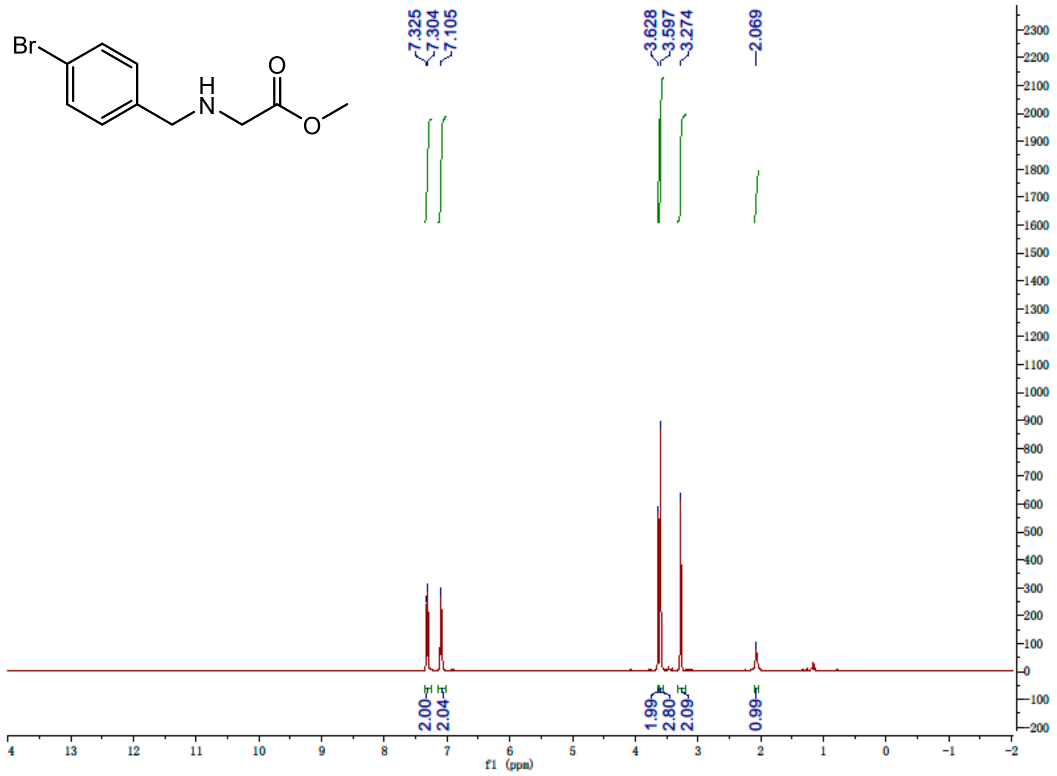

Supplementary Figure S35. ^{1}H NMR spectra of compound **3s** ($CDCl_3$).

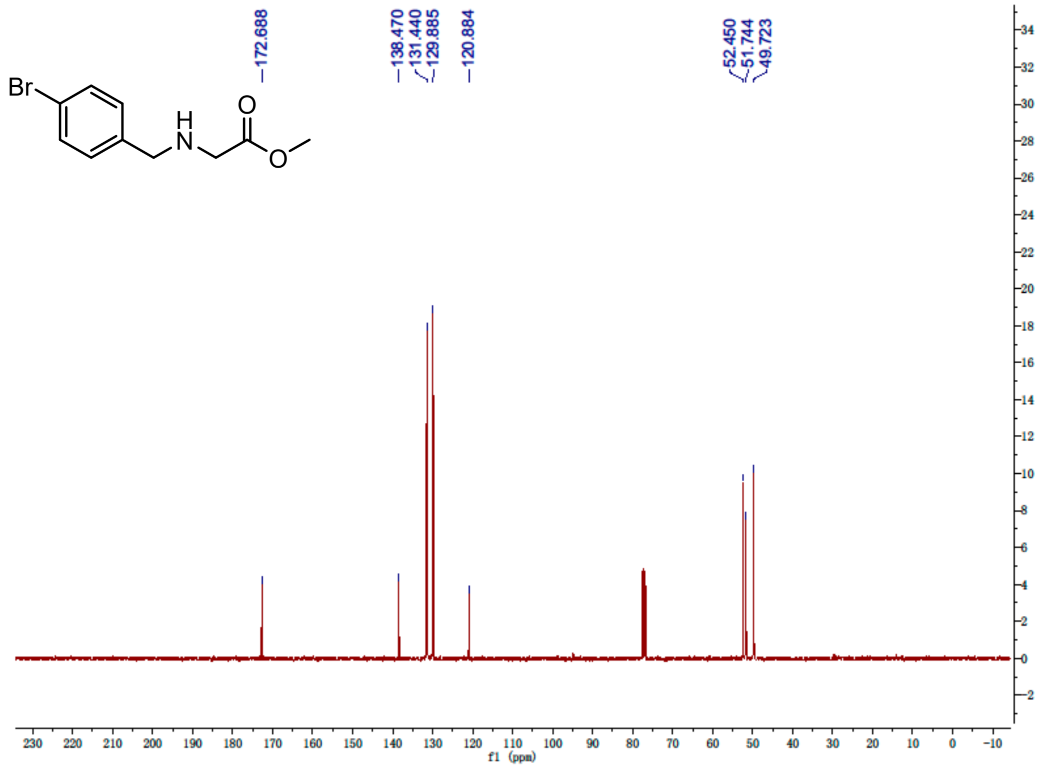

Supplementary Figure S36. ^{13}C NMR spectra of compound **3s** ($CDCl_3$).

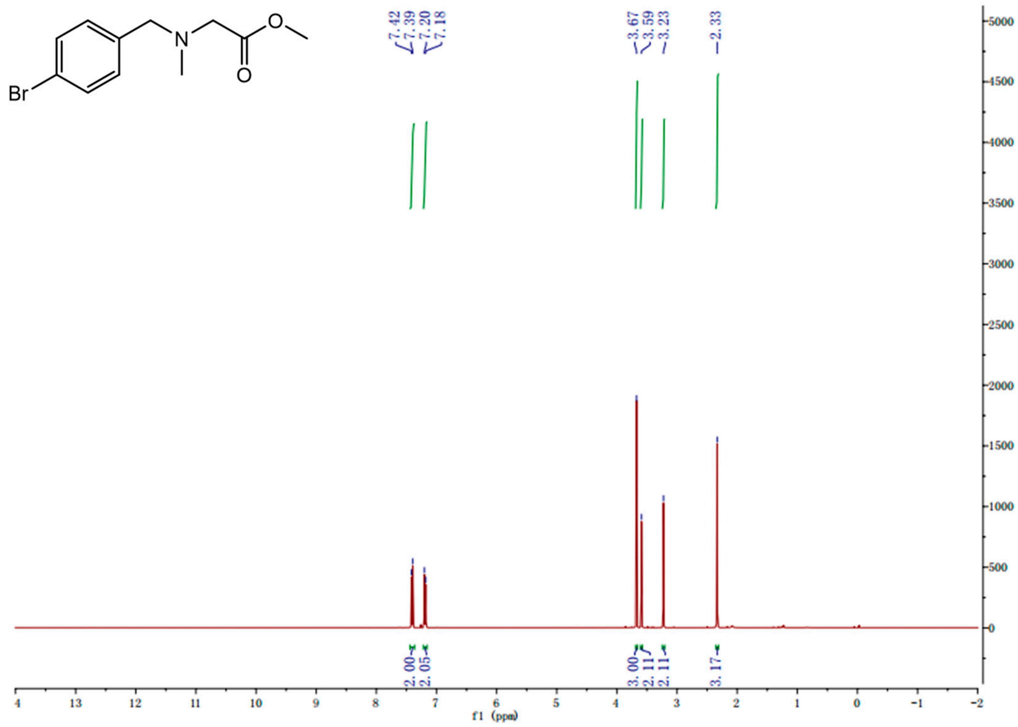

Supplementary Figure S37. ^{1}H NMR spectra of compound **4s** ($CDCl_3$).

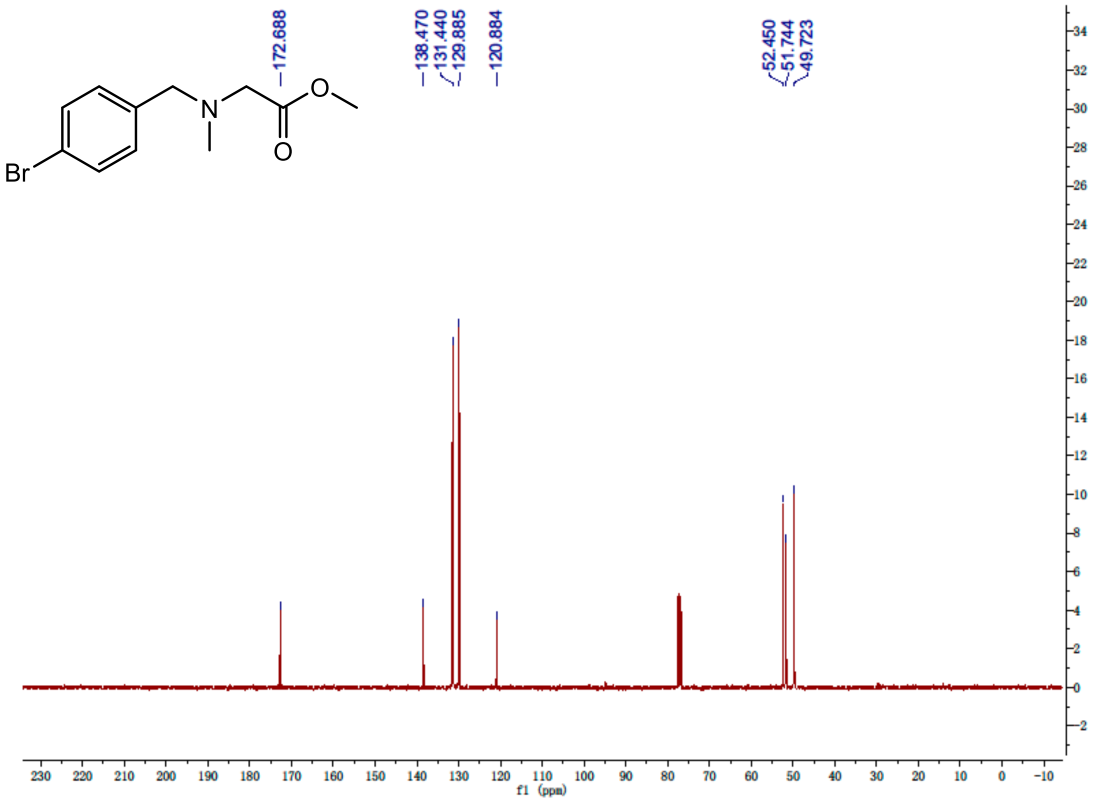

Supplementary Figure S38. ^{13}C NMR spectra of compound **4s** ($CDCl_3$).

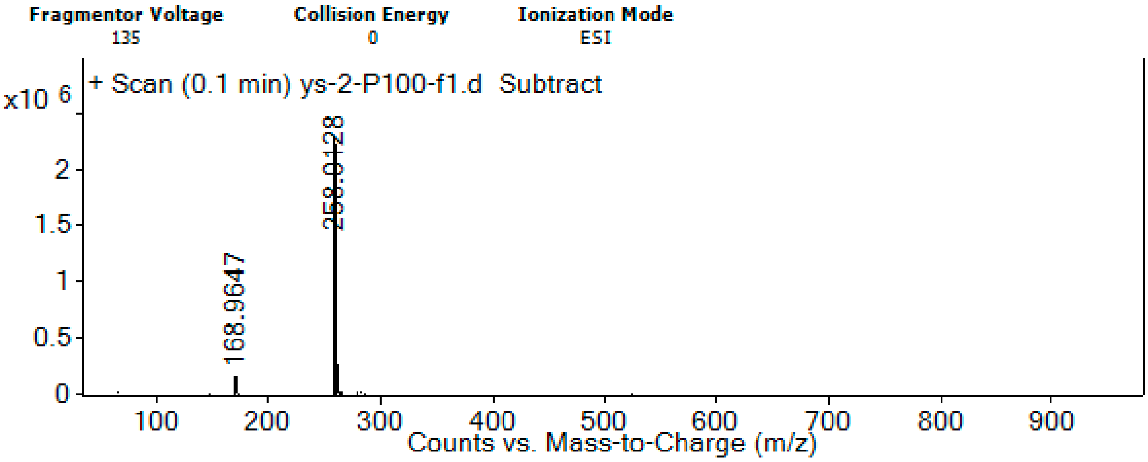

Supplementary Figure S39. HRMS (ESI-TOF) spectra of compound **5s**.

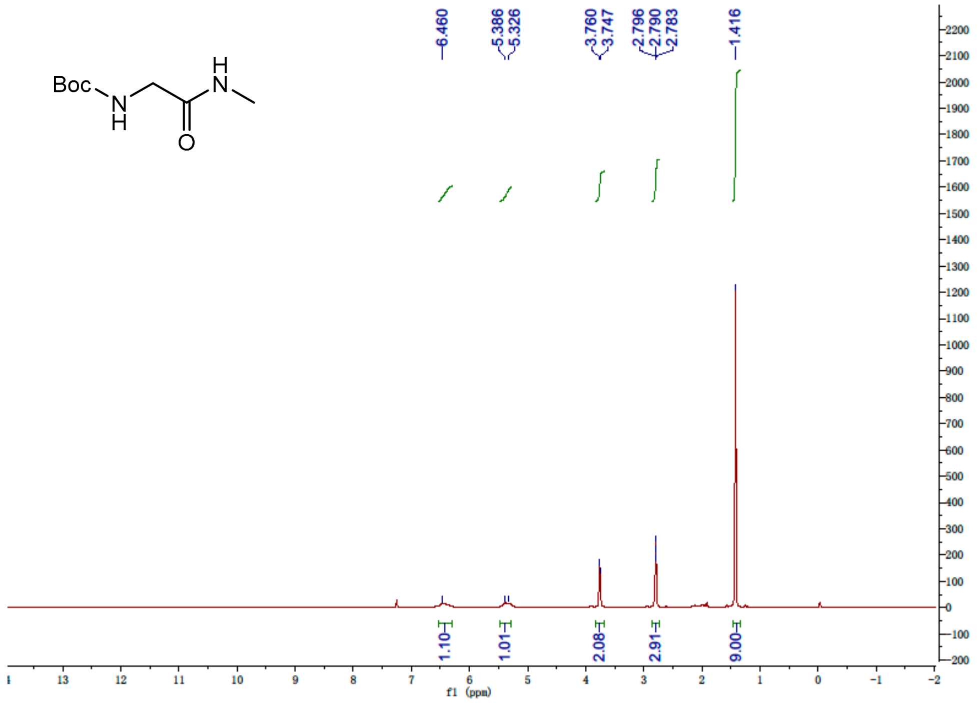

Supplementary Figure S40. ^{1}H NMR spectra of compound **6s** ($CDCl_3$).

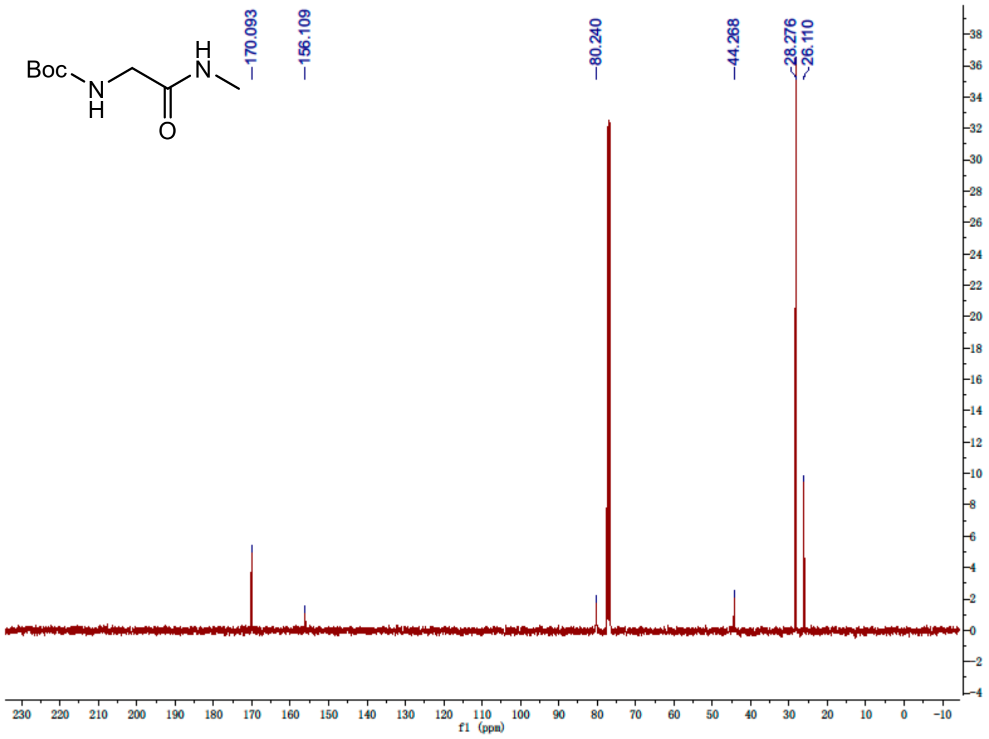

Supplementary Figure S41. ^{13}C NMR spectra of compound **6s** ($CDCl_3$).
